# The strong arm of the law: a unified account of necessary and contingent laws of nature

**DOI:** 10.1007/s11229-021-03243-z

**Published:** 2021-06-29

**Authors:** Salim Hirèche, Niels Linnemann, Robert Michels, Lisa Vogt

**Affiliations:** 1 Département de philosophie, Université de Genève, 2 rue de Candolle, 1211 Geneva 4, Switzerland; 2 Institut für Philosophie, Universität Bremen, Postfach 330 440, Enrique-Schmidt-Str. 7, 28334 Bremen, Germany; 3 Rotman Institute of Philosophy, University of Western Ontario, Western Interdisciplinary Research Building, 1151 Richmond Street North, London, ON N6A5B7 Canada; 4 Facoltà di scienze della comunicazione, Università della Svizzera italiana, Via G. Buffi 13, 6900 Lugano, Switzerland; 5 Institut für Philosophie, Universität Bern, Hochschulstrasse 6, 3012 Bern, Switzerland; 6 Facultat de Filosofia, Universitat de Barcelona, LOGOS, Carrer de Montalegre 6-8, 08001 Barcelona, Spain; 7 Institut für Philosophie, Freie Universität Berlin, Habelschwerdter Allee 30, 14195 Berlin, Germany; 8 Theologische Fakultät, Universität Luzern, Frohburgstrasse 3, 6002 Luzern, Switzerland

**Keywords:** Laws of nature, Metaphysical necessity, Nomic necessity, Necessitarianism about laws of nature, Kinematical/dynamical distinction

## Abstract

A common feature of all standard theories of the laws of nature is that they are ‘absolutist’: They take laws to be either *all* metaphysically necessary or *all* contingent. Science, however, gives us reason to think that there are laws of both kinds, suggesting that standard theories should make way for ‘non-absolutist’ alternatives: theories which accommodate laws of both modal statuses. In this paper, we set out three explanatory challenges for any candidate non-absolutist theory, and discuss the prospects of the two extant candidates in light of these challenges. We then develop our own non-absolutist theory, the essentialist DTA account, which combines the nomic-necessitation or DTA account with an essentialist approach to metaphysical modality in order to meet the three explanatory challenges. Finally, we argue that the distinction between kinematical and dynamical laws found in physical theories supports both non-absolutism in general and our proposed essentialist DTA view in particular.

## Non-absolutism about laws

### Why be a non-absolutist?

A central question in the metaphysics of science is what a law of nature is. In the recent literature, three philosophical theories of laws of nature have established themselves as standard theories: *Dispositional essentialism*, the *Dretske-Tooley-Armstrong* (*DTA* for short) or *nomic necessitation view*, and *Humeanism*.^1^

A crucial point of disagreement between these theories concerns the modal status of the laws. Dispositional essentialism tells us that the laws of nature are metaphysically necessary, that is, necessary in a very strong sense, whereas the nomic necessitation view and Humeanism both tell us that they are metaphysically contingent. One thing which all three theories have in common, however, is that they are *absolutist*: They attribute one and the same modal status to all laws of nature.^2^

Yet, a closer look at actual examples of physical laws casts doubts on this absolutist dogma: On the one hand, certain laws simply do not seem sensible candidates for having the status of metaphysical necessities. Among others, Tahko (2015) has pointed out that a change in the fine-structure constant over time reported by physicists allows us to infer its modal variance and thereby the metaphysical contingency of laws involving this constant. In a similar spirit, Hendry and Rowbottom (2009) have argued that certain scientific explanations require counternomic possibilities, possible situations ruled out by at least some laws of nature, questioning the assumption that all laws are metaphysically necessary. Moreover, it is hard to see for many law-like statements as to why they should hold with metaphysical necessity. Just take Coulomb’s law: Why think that the force between two electrons could not in any objective sense have been different? There are thus what appear to be good reasons to assume, contra dispositional essentialism, that certain physical laws are not metaphysically, but rather only nomically necessary.

On the other hand, there are physical laws which do after all seem to hold with metaphysical necessity. An example usually provided (also by Tahko 2015) is that of the Pauli exclusion principle (PEP). The PEP states that no two fermions in a closed system can occupy the same quantum state at once. According to Tahko, the principle can be seen to hold in virtue of the nature of fermions.^3^ This would make the PEP an example of a metaphysically necessary law, as long as one presupposes the commonly held view that propositions *which hold in virtue of the nature of something* express metaphysical necessities. (Cf. Tahko 2015, p. 524-5.) In fact, it seems that the PEP qua law-like statement is just so centrally constitutive of fermions that we could take it to specify a constraint on any admissible way our universe could objectively have been in the presence of fermions, giving us another reason to think that it holds with metaphysical necessity.^4^

Hence, there are reasons to explore non-absolutist alternatives to the standard theories, that is, theories of the laws of nature which allow for both metaphysically contingent, as well as metaphysically necessary laws of nature. This paper contributes to this project, but it also adds its own twist to it. Existing arguments for non-absolutism are formulated in a naturalistic spirit. They in particular rely on relevant scientific knowledge in order to establish a philosophical conclusion. This paper is written in a similar spirit, but unlike Tahko and Hendry and Rowbottom, our reliance on science goes beyond that of treating it as a source for counterexamples to absolutism. We follow them in proposing a non-absolutist theory of the laws which is at its core purely philosophical. We also agree with their assessment that Coulomb’s law and the PEP are examples of metaphysically contingent and necessary laws, respectively. Yet, we also argue that there is an established physical distinction, the distinction between kinematical and dynamical constraints, which can, in conjunction with philosophical considerations, (a) serve as a motivation for non-absolutism about the laws of nature, thus giving us a more systematic, less intuition-dependent motivation for non-absolutism, and (b) provide a criterion for the metaphysical necessity of certain laws. Physics thus contributes more to our constructive philosophical proposal than it did in previous works on non-absolutism; and, in this sense, our paper could be called more naturalistic.

In the rest of this section, we will first identify three main explanatory challenges that any non-absolutist view of the laws has to address (Sect. 1.2). We will then argue that the two main available proposals for non-absolutist views, Tahko’s and Hendry and Rowbottom’s, fail to meet at least some of them or require controversial assumptions about properties, respectively (Sect. 1.3). Based on these considerations, we will then develop our own proposal for a non-absolutist view—a modified version of the standard DTA-view, according to which the relevant relation of nomic necessitation between the universals involved in a law may hold either metaphysically contingently (as it does according to the standard account) *or* metaphysically necessarily. As we will argue, this account can meet all of the three metaphysical challenges in a natural and straightforward way (Sect. 2). Finally, we will further develop our proposed non-absolutist view by showing how it can, in addition, meet the *epistemological* challenge of providing a reliable, scientifically informed criterion to determine which laws are metaphysically necessary and which laws are metaphysically contingent. Our proposed criterion will rely on the kinematical/dynamical distinction, which is familiar from physical theorizing (Sect. 3). We will end with some concluding remarks (Sect. 4).

### What a non-absolutist theory should explain

It is a common feature of the main absolutist theories of laws of nature that their explanations of what a law of nature is also fixes whether the law is metaphysically necessary or contingent. Dispositional essentialists hold that laws of nature describe the causal powers of things. This implies that all laws are metaphysically necessary, given that powers are essentially connected to the causal roles that they play, and that all entities possess their essences with metaphysical necessity. (See e.g. Bird 2007; Ellis 2001.) According to the DTA view, or nomic necessitation view, laws of nature are only nomically, but not metaphysically necessary, since they capture a nomic necessitation relation which metaphysically contingently holds between universals. (See e.g. Armstrong 1983; Dretske 1977; Tooley 1977). Finally, Humeanism also tells us that laws of nature are metaphysically contingent. According to Humeans, laws of nature are axioms of our best systematization of all the non-modal facts about the world, and these facts could (in the metaphysical sense) have been different. (See e.g. Lewis 1973.)

Compared to these theories, a non-absolutist theory which admits both metaphysically contingent *and* metaphysically necessary laws faces particular explanatory challenges. First, it has to account for *two* sorts of necessity with which the laws may hold: metaphysical necessity and nomic necessity. For, once metaphysical necessity has been accounted for, nomic necessity does not just come for free. Unlike mere accidental generalizations, which are also metaphysically contingent, metaphysically contingent *laws* are still supposed to be nomically necessary. And this other form of necessity also needs to be explained. Thus, a non-absolutist view should really provide us with two explanations: It should tell us for both metaphysical and nomic necessity where these kinds of necessity find their source. Needless to say, this explanation should be a *plausible* one. In particular, the view should make it clear why the proposed source is relevant to the kind of necessity considered, and it should not yield counterintuitive results regarding the extent of that kind of necessity—e.g. it should not entail that any true or false proposition whatsoever has that modal force.

Second, the respective explanations of metaphysically and nomically necessary laws provided by a non-absolutist theory should be ‘synchronized’, in the sense that laws should not be attributed conflicting modal statuses. In particular, the theory should ensure that both metaphysically contingent *and* metaphysically necessary laws are always nomically necessary. This second challenge mainly concerns the *relation* between nomic necessity and metaphysical necessity, and more specifically how their respective *extents* harmonize.

It is worth expanding a bit on this challenge, which we may call ‘the synchronization challenge.’ How can a non-absolutist theory address it? Let us first consider a theory which posits two distinct sources for the two sorts of modality. Generally, if these sources are genuinely independent, then there seems to be no direct way for the theory to ensure that they assign harmonious modal statuses to a law (or to any related proposition more generally): If the two sources are genuinely independent, then that means that the modal status which is assigned to a law by one source is not in any way constrained by the modal status assigned to it by the other source. To ensure that the two sources are in sync, one would thus need to posit a third factor which has the power to constrain both sources of modality. Plausibly, this third factor would need to be modal too—it could perhaps consist of a logical (meta-)modality which ensures the logical consistency of the modal statuses assigned to laws by the two sources. This, however, raises the question of why this third kind of modality has a constraining power over other sources of modality.

Difficult questions of this sort are avoided by non-absolutist theories which do not posit two distinct sources of modality, but rather claim that one kind of modality which applies to the laws can actually be understood in terms of, or derived from, the other. According to such a theory, there is, fundamentally, only one source of modality. The most straightforward way to implement this idea in the context of a non-absolutist theory is to think of the nomic possibilities as a proper subset of the metaphysical possibilities. If one’s account yields this result, the two kinds of modality cannot conflict in the described way, because each nomic possibility is also a metaphysical possibility, which means that metaphysical necessity implies nomic necessity.^5^

Generally, it seems that an answer to the synchronization challenge is much easier to come by if one adopts a one-source view. A non-absolutist theory based on such a view may e.g. explain all modality in terms of truth in possible worlds, in terms of essences, or of powers. It is much less clear whether, and if so, how a theory based on a two-source view of the two kinds of modality could guarantee that no multi-modal incoherences arise.

Third, since non-absolutist theories of laws draw a line between two distinct classes of laws—those which are metaphysically contingent and those which are metaphysically necessary—there is a risk that such a theory may fail to deliver a unified account of what all laws of nature, whatever their modal statuses, have in common, i.e. of what, *in general*, makes a law a law. Here is how Bartels (2019) expresses the worry:

...this move [to a non-absolutist theory of laws] would then require, first of all, some different demarcation criterion of lawhood (with respect to empirical regularities) which would be independent from metaphysical necessity/contingency, i.e. we should be able to know, whether some empirical truth is a law of nature or not, independently of whether it holds by metaphysical necessity or not. (Bartels 2019, p. 10)

Bartels’ requirement seems justified: There should be a common account of lawhood which is independent of modal status, in the sense that it goes beyond the trivial stipulation that the laws are those law-like statements which are nomically (or metaphysically) necessary, and, one may add, which is not merely disjunctive. The need to provide such an account seems particularly pressing given a non-absolutist view of the laws.

In sum, a non-absolutist view of the laws faces three specific challenges:

1. *The double explanatory challenge*: The view should account for both of the potential modal statuses of the laws, i.e., metaphysical necessity and nomic necessity.
2. *The synchronization challenge*: The view should exclude inadmissible combinations of metaphysical and nomic modal statuses of a law, such as a metaphysically necessary law’s being nomically contingent.
3. *The common lawhood criterion challenge*: The view should provide a *non-disjunctive* account of lawhood which does not simply identify a law of nature with a nomically or metaphysically necessary generalization.

With that being said, we will now turn to the two non-absolutist theories of laws that have been proposed in the recent literature, and discuss whether they can meet those three challenges.

### Existing proposals and their problems

#### Tahko’s hybrid view

Tahko (2015) contains, to the best of our knowledge, the first suggestion of an explicitly non-absolutist theory of the laws proposed in the literature. While the paper does not develop the theory in enough detail to directly assess its ability to meet the three challenges set out in the previous section, it is worth discussing the theory’s core idea and how it could be further developed to meet the challenges.

Tahko clearly indicates what he takes to be the source of metaphysical necessity for any laws of this status. The core assumptions of his view are that a law of nature is metaphysically necessary if it features a fundamental natural kind and that it is metaphysically contingent if it does not. (See Tahko 2015, p. 518.) According to Tahko, the PEP, for example, has the former modal status, since it expresses an essential truth about the kind *fermion*, whereas Coulomb’s law lacks this status, since there is no plausible candidate for a natural kind whose essence is captured by this law.^6^

Tahko is, however, less clear about why both metaphysically necessary and contingent laws are still nomically necessary. He approvingly refers to Birds’ description of Armstrong’s idea that nomic necessities are ‘soft’, or in Tahko’s preferred terminology, ‘weak’ necessities, which are characterized in terms of their explanatory force and ability to support counterfactuals. (See Bird 2005, p. 148.) As Tahko points out, this characterization naturally aligns with a Humean view of the laws, but he also mentions, without going into any detail, the possibility that the nomic necessity of the laws may be explained in terms of them featuring natural properties, as opposed to natural kinds. (See Tahko 2015, p. 519.) In the following, we will briefly discuss how Tahko’s hybrid view could be further developed along either of these two lines and argue that neither developement results in a non-absolutist theory which is able to meet the explanatory challenges mentioned in Sect. 1.2.

We can be relatively brief with the first suggestion, viz., that of relying on a Humean account of the laws to explain their nomic necessity. The resulting theory would both be hybrid in the sense that it allows for both metaphysically necessary and contingent laws (this is Tahko’s intended sense of ‘hybrid’) and in the sense that it involves two distinct sources of modality, the essences of natural kinds and the status of being axioms in the best systematisation of all scientific truths about the actual world.^7^ So understood, the view meets the first explanatory challenge since it specifies the sources of metaphysical and nomic necessity.

As we have pointed out earlier, a non-absolutist theory which posits two genuinely independent sources of modality will likely have difficulties guaranteeing modal harmony between the nomic and metaphysical modal status of a law. The doubly-hybrid version of Tahko’s theory is a case in point. If we assume that a particular law involves a natural kind in the sense envisioned by Tahko, then this does not at all guarantee that the law is also an axiom (or theorem) of the best system (nor does the converse hold). There is no good reason to think that the theory-intrinsic aspects responsible for making a law an axiom of the Humean best system (usually assumed to be: simplicity, fit, and explanatory strength) harmonize with a theory-independent, purely ontological inventory of kinds. Hence, the doubly hybrid theory does not meet the second challenge.

And, finally, given that the doubly hybrid theory posits two fundamentally different kinds of laws—those flowing from the essences of fundamental natural kinds and those which are axioms of the best system—it is at best unclear how it could meet that third challenge, viz. that of providing a non-trivial common criterion for lawhood.

Let us now briefly consider Tahko’s second suggestion, namely that some laws are metaphysically necessary because they involve a natural kind, whereas others are metaphysically contingent, but still nomically necessary, because they merely involve a natural property instead. The problem with this second proposal is that it is not at all clear how the distinction between natural kinds and natural properties is supposed to make a difference regarding the modal status of the laws. After all, Tahko’s view tells us that the metaphysical necessity of the laws featuring natural kinds is due to their essences, which natural properties presumably also have. But essence cannot do the trick, since essentiality implies metaphysical, not mere nomic necessity, no matter whether the relevant essence is that of a natural kind or a natural property. Relying on essence as the source of nomic necessity would thus turn the theory into an absolutist view, according to which all laws are metaphysically necessary. Alternatively, one might posit a second source of necessity distinct from essence in order to account for the nomic neccessity of the laws. But this sort of account would then bring us back to the same problems which plague the doubly-hybrid version of the hybrid theory.

To sum up, we do not see how either one of Tahko’s two suggestions could yield a non-absolutist theory which successfully meets all the explanatory challenges for non-absolutist accounts.

#### Hendry and Rowbottom’s dispositional contextualism

The non-absolutist theory of Hendry and Rowbottom (2009) is a more permissive version of dispositional essentialism called *dispositional contextualism*. They motivate their theory by arguing that there are legitimate scientific explanations which involve counternomic possibilities, i.e., possible worlds in which some laws fail to hold. Standard dispositional essentialists cannot admit such possibilities, since according to them, laws of nature are metaphysically necessary, that is necessary in the strongest sense, which means that there are no counternomic possibilities.

Like standard dispositional essentialists, Hendry and Rowbottom subscribe to an anti-quidditist account of properties according to which properties play their nomic roles essentially. According to this account, something can, for example, only be water if it has the same dispositional profile as actual water. If implemented in the way that standard dispositional essentialism does, anti-quidditism implies that the laws of nature are metaphysically necessary: Properties possess their dispositional profiles with metaphysical necessity, laws of nature capture these profiles, and therefore, the laws of nature turn out to be metaphysically necessary—that is, they hold in all metaphysically possible worlds.

Hendry and Rowbottom resist this implication by adopting a non-standard view of properties, which provides the central building block of their dispositional contextualism. According to this view, dispositional properties have two particular features: they are (i) context-sensitive and (ii) vague. That they are context-sensitive implies that ‘having a property *P* may involve manifesting *M* in response to *S* in some contexts, but manifesting *M*$_1$ in response to *S* in other contexts, where such contexts are possible worlds.’ (Hendry and Rowbottom 2009, p. 673.) According to dispositional contextualism, dispositional properties really consist of the different dispositional profiles which the objects with this property exhibit relative to different possible worlds. This means that they are cluster properties, collections of world-bound properties, each of which has a dispositional profile in its respective world. What ties the world-bound properties together are counterpart-relations, i.e. cross-world relations of similarity between them. (Cf. Hendry and Rowbottom 2009, p. 674.) In case of the water-property, for example, the bundle would consist of an actual-world-water-property which has water’s dispositional profile with respect to the actual world (which would be complex, capturing all of water’s actual dispositions) and of one world-relative water property for each possible world—some of which according to Hendry and Rowbottom, differ from the actual-world-water-property. Hendry and Rowbottom’s idea is that this allows water to have a different dispositional profile relative to some possible worlds. For instance, in some other possible world, the dispositional profile of water might omit the disposition of actual $\hbox {H}_2$O molecules to enter into hydrogen bonds with other molecules containing highly electronegative atoms, including other $\hbox {H}_2$O molecules. To pick up their example, assuming that it is an actual law of nature that hydrogen bonding occurs,^8^ this law will not hold in such a world, since it does not apply to $\hbox {H}_2$O molecules in that world.

But what allows a dispositional contextualist to claim that in such a possible world, water is still water, even though it fails to display (parts of) its characteristic dispositional behaviour? This is where the vagueness of the properties comes into play.

Dispositional cluster properties consist of a set of world-relativized dispositional profiles and counterpart-relations which, so to speak, glue them together. These relations play the same role in Hendry and Rowbottom’s theory as they do in their original context, Lewis’s metaphysics of modality:^9^ They provide one with something similar, but not quite the same as numerical identity. All the core differences to identity can be traced to the fact that counterparthood is a matter of overall similarity between objects; in the case of dispositional contextualism, these objects are dispositional profiles, or the associated world-relative properties. Whether a dispositional profile (or the associated property) and another are counterpart-related is a matter of how similar they are. Hence, water, conceived of as a dispositional cluster property, can have a dispositional profile which deviates from water’s actual dispositional profile regarding e.g. its boiling point relative to a counternomic, but metaphysically possible world, if that profile is still overall similar enough to water’s actual dispositional profile for the former to be a counterpart of the latter. The price to pay for this flexibility is that of it being a vague matter when a world-relative dispositional profile stops being overall similar enough to qualify as a counterpart of e.g. the dispositional profile of water in the actual world. (Cf. Hendry and Rowbottom 2009 p. 676.)^10^

Dispositional contextualism is designed to account for the metaphysical possibility of counternomic worlds and correspondingly also for the metaphysical contingency of some laws of nature. Metaphysically contingent laws are laws which capture the dispositional profiles of the relevant property in some possible worlds, but not in others.^11^ The theory can furthermore easily account for the metaphysical necessity of some laws of nature: The property underlying such laws has a constant dispositional profile relative to all metaphysically possible worlds.^12^

Can dispositional contextualism meet the explanatory challenges which we raised for non-absolutist theories of laws of nature? To answer the question, we have to go beyond the suggestive, but rather piece-meal theory Hendry and Rowbottom present. In particular, we need to specify how dispositional contextualism handles nomic necessity. We will do so by supplementing the theory with the standard view that nomic necessity amounts to truth in all nomically possible worlds, where a nomically possible world is one in which the same laws of nature hold as in the actual world. Speaking in terms of Hendry and Rowbottom’s cluster properties, these worlds are just those for which a relevant class of cluster properties (e.g. made relevant by their fundamentality) contain the same dispositional profile as they do for the actual world.

With this account of nomic necessity in hand, we can now take stock. Dispositional contextualism can indeed meet the first challenge, since it provides an account of both metaphysically necessary laws and merely nomically necessary laws: metaphysically necessary laws express truths about dispositional properties which have a constant dispositional profile across all metaphysically possible worlds; merely nomically necessary laws express truths about dispositional properties which have a dispositional profile which is constant across all nomically possible worlds, but varies in counternomic metaphysically possible worlds.

Dispositional contextualism is also able to meet the synchronization challenge, i.e. the second challenge, since it presumably posits only one source of necessity for the laws, the essences of the dispositional properties which give rise to them. In particular, the nomic necessity of any metaphysically necessary law is guaranteed by the fact that the underlying dispositional cluster property exhibits a constant dispositional profile across all metaphysically possible worlds: a dispositional profile of this kind holds also throughout all nomically possible worlds, since they are a subset of the metaphysically possible worlds.

What about the third challenge—that of providing a common, non-disjunctive criterion for lawhood which is independent of modal status? Since the theory is a variant of dispositional essentialism, one natural idea would be to rely on the (dispositional) essences of properties to provide such a criterion. Following this line of thought, one might, for example, say that the PEP is a law because it expresses an essential truth about the property of being a fermion. However, while this criterion may work well for dispositional essentialists, dispositional contextualists would face serious problems if they relied on it.

First, it is unclear whether talk of essences of dispositional cluster properties even makes sense, since they are mere bundles of pairwise similar world-relative dispositional profiles held together by counterpart relations. What could the essence of such a bundle be, given that the bundle may encompass very different dispositional profiles across worlds? Since it is not clear how dispositional contextualists should answer this question, it seems that they cannot simply rely on the lawhood criterion provided by dispositional essentialism.

Second and even more importantly, the dispositional essentialists’ lawhood criterion would immediately turn Hendry and Rowbottom’s theory into an absolutist view. Unlike dispositional essentialists, dispositional contextualists also have to account for metaphysically contingent laws—a task for which essence seems simply unfit: since essentiality entails metaphysical necessity, any lawlike generalization which expresses an essential truth about a property thereby also expresses a metaphysical necessity.

In order to avoid these problems and to make room for metaphysically contingent laws, dispositional contextualists may adopt a modified lawhood criterion, based on a different take on the essences of their dispositional cluster properties: The starting point would be the claim that what is essential to the relevant dispositional cluster property is not a single, general, and world-independent dispositional profile which could directly ground a law. Rather, the idea is that the property has *all* of its *world-relative* dispositional profiles essentially: For instance, it would then be essential to water that it has its boiling point of 100$^{\circ }$C (ceteris paribus) relative to the actual world, and that it has a certain lower boiling point of say, 80$^{\circ }$C (under the same conditions), relative to a particular counternomic world, and so on for all the other possible worlds. Accordingly, what is rendered metaphysically necessary by a dispositional cluster property’s essence is not directly a law, but rather the fact that this property has these dispositional profiles *relative* to the relevant worlds. As a result, the drawback of relying on essence mentioned above is avoided: a law-like statement does not have to be metaphysically necessary anymore to be a law. For a true generalization to be a law, it suffices that it is essential to that property that it has a pertinent dispositional profile relative to the *actual* world—which is compatible with it being essential to that property that it has a different profile relative to some other possible worlds.

Although that modified lawhood criterion, unlike the standard dispositional essentialist criterion, avoids the consequence that all laws are metaphysically necessary, it still faces a problem, namely that it makes it unclear what role essence would even play in the account. For according to it, the essence of such a property fixes the whole set of world-relative dispositional profiles which belong to the property, which means that all the work in settling the modal status of the laws is already done at this point. If a property has a dispositional profile which is constant relative to all worlds, the corresponding law will be metaphysically necessary; if not, the law will be merely nomically necessary. It is then unclear how essence is supposed to contribute to accounting for the modal status of laws.

In that respect, dispositional contextualism, based on the lawhood criterion under consideration, would be very different from standard dispositional essentialism—arguably more so than what Hendry and Rowbottom suggest. According to standard dispositional essentialism, what is essential to the relevant property is a specific dispositional profile; and that profile holds in all possible worlds *because* it is essential (absolutely, not relative to some worlds) to the property to have that profile. Thus, essence clearly accounts for the modal status of the law: It is metaphysically necessary because it is essential to the property. But on the picture suggested by the modified criterion, things are quite different: A property already encodes all of its world-relative dispositional profiles; and those already tell us whether a law will be metaphysically necessary or not. The only further modal role which essence could play in this picture is that of accounting for a ‘second-order’ modality: The fact that this set of world-relative profiles is *essential* to the relevant property may be taken to account for the fact that, whatever modal status this set gives to a law (metaphysical necessity or mere nomic necessity), the law has that modal status essentially, hence with metaphysical necessity—i.e. it is metaphysically necessary that the law is metaphysically/nomically necessary. But explaining the necessity of the necessity of a law is evidently a far cry from explaining why the law is a law in the first place.

In sum, it seems that a lawhood criterion based on the essence of properties would be problematic for dispositional contextualists. However, the previous discussion suggests that they could opt for a criterion for lawhood which is not based on essence at all. Not only would this allow them to avoid the problems just mentioned, but more generally, getting rid of essence would be a quite natural move considering the apparent mismatch between the highly context-dependent and flexible counterpart relation *on which their view decisively relies*, on the one hand,^13^ and the absolute and rigid notion of essence usually posited by contemporary essentialists (and in particular by dispositional essentialists), on the other hand.

The idea would be to simply say that laws are true generalizations that are due to some property, in particular to its whole set of world-relative dispositional profiles. More specifically, for a generalization to be a law, it has to be the case that the generalization expresses a dispositional profile (e.g. boiling at 100$^\circ$C under normal conditions) that a property (e.g. water) has relative to the actual world.^14^

An essence-free criterion of this sort appears to provide dispositional contextualists with an acceptable answer to the third explanatory challenge for non-absolutist views, giving them a lawhood criterion which is non-disjunctive and common to all laws of nature, whether metaphysically necessary (supported by a dispositional cluster property with a dispositional profile which is constant through all possible worlds) or merely nomically necessary (supported by a dispositional cluster property which is constant throughout some worlds).

We have just argued that, after a bit of work, dispositional contextualism is able to meet all three explanatory challenges for non-absolutist theories. Still, there are good reasons to search for alternatives. Dispositional contextualism stands and falls with Hendry and Rowbottom’s non-standard account of dispositional properties. This account is not likely to find broad acceptance among philosophers. For one, it requires one to accept that there are vague properties. This is a highly controversial claim which conflicts with the widely accepted view that ‘[t]he only intelligible account of vagueness locates it in our thought and language.’ (Lewis 1986, p. 212.)^15^ As we have just argued, in order to meet the third explanatory challenge, dispositional contextualists may also have to strip their account of a core ingredient of dispositional essentialism, namely the essences of dispositional properties, even though the former was supposed to offer a non-absolutist alternative to the latter theory. Where should philosophers who have sympathies for non-absolutism, but prefer a more conservative metaphysics of properties turn then? Tahko’s view does not meet all of the the explanatory challenges, so what is the alternative?

## The essentialist DTA account of laws

Since one of the two extant non-absolutist theories of the laws of nature does not meet the explanatory challenges and the other relies on a controversial view of properties, we should search for alternatives. The aim of the remainder of the paper will be to propose and further explore such an alternative. In this section, we will focus on the purely metaphysical aspects of the proposal, viz., on laying out an account of the laws of nature which makes room for non-absolutism about the modal status of the laws and which meets the explanatory challenges faced by any non-absolutist view. In the following section, we will then broaden the focus to the epistemological question of how we can find out whether a law is necessary or contingent. For this, we shall develop a naturalist criterion for detecting the modal status of a given law which naturally complements the metaphysical account suggested in this section.

Our account takes its point of departure from one of the standard accounts of laws in the literature, viz., the *nomic necessation view*, which is also called the ‘DTA theory’ after its main defenders Armstrong (1983), Dretske (1977, 1987) and Tooley (1977). Our proposed account modifies the DTA theory in two major respects in order to make room for metaphysically contingent and necessary laws and we will also argue that it can solve a major problem which the original theory faces. We begin with an outline of the standard version of the theory (Sect. 2.1), go on to present our own version of the account (Sect. 2.2), and finally show how it can meet the explanatory challenges for non-absolutist views (Sect. 2.3).

### The standard DTA account

The core idea of the DTA account is that laws of nature are due to a second-order relation of nomic necessitation—or, for short, “${\mathcal {N}}$-relation”—that holds between universals.^16^ Whether properties stand in this relation is taken to be a matter of contingency. That is, some property ${\mathcal {F}}$ may stand in the relation to ${\mathcal {G}}$ in our world,^17^ and yet fail to stand in it in some other world:

- Contingent Holding: $$N({\mathcal {F}}, {\mathcal {G}}) \rightarrow \diamond \lnot N({\mathcal {F}}, {\mathcal {G}})$$.

The proponent of the DTA account then takes the instantiations of ${\mathcal {N}}$ to be intimately connected to first-order regularities regarding the related universals in the following way: Whenever ${\mathcal {F}}$ stands in ${\mathcal {N}}$ to ${\mathcal {G}}$, all *F*s are *G*s:

- Implication: $$N({\mathcal {F}}, {\mathcal {G}}) \rightarrow \forall x (Fx \rightarrow Gx)$$.^18^

The claim about a material implication between the holding of ${\mathcal {N}}$-relations and the corresponding generalizations raises the question of the modal robustness of the implication: Do we encounter this implication only in our world, or in others as well? And, in particular, does the implication hold in all possible worlds? The latter claim would amount to:

- Necessary Implication: $$\Box (N({\mathcal {F}}, {\mathcal {G}}) \rightarrow \forall x (Fx \rightarrow Gx))$$.

(Necessary Implication) is a very natural claim to endorse given (Implication). Do the three main proponents of the standard DTA account actually endorse (Necessary Implication)? Tooley holds that the conditional is logically necessary, which would imply its metaphysical necessity. Things are slightly less clear with Armstrong and Dretske. Armstrong (1983) maintains that ‘it seems that we will have to say that the entailment holds in virtue of a *de re* necessity linking the relation between the universals, on the one hand, and the uniformity it ‘produces’, on the other.’ (p. 86). But at the same time, he claims that ${\mathcal {N}}$ is a ‘relation of *contingent* necessitation’, which might be read as a denial of the principle. Finally, while the analogies that Dretske (1977) draws between the workings of ${\mathcal {N}}$ and legal codes suggest that he endorses (Necessary Implication) (cf. p. 264), he does not explicitly commit to it.

No matter, however, whether it is maintained that we have this relationship of entailment (i.e. *necessary* implication) or not, the laws of nature will turn out to be metaphysically contingent according to the original DTA account. Given that it is a matter of contingency whether the relevant properties stand in the *N*-relation in the first place, it follows that it is a matter of contingency whether the corresponding generalizations obtain. The original DTA account is thus clearly a contingentist absolutist view.

There are arguably two major challenges for the DTA account, the *form of the law problem* and the *inference problem*.^19^

Let us start with the form of the law problem. This problem departs from the observation that, on the face of it, (virtually) none of the laws as described by current physics seem to match the ‘all *F*s are *G*s’-pattern. In particular, physical laws typically describe functional relationships between values of properties. This point seems rather obvious and can already be acknowledged without having to rely on much empirical consideration; still, it is arguably best seen through actual examples of laws from current physics. For instance, take our previous two main examples: The PEP has it that no two fermions can have the same quantum numbers in a closed system, and Coulomb’s law has it that the electrostatic components of force between two electrons is proportional to the product of each other’s charge according to the equation $F_Q= F_q = \frac{1}{4 \pi \epsilon _0} \frac{Q q}{r^2}$. None of them looks, on the face of it, like having the form ‘All *F*s are *G*s’. But if laws of nature like these do not fit this pattern, then the DTA account would seem to be inadequate, or so the worry goes.

This topic has sparked much controversy, with many options outlined in the literature and no stable agreement reached.^20^ Offering a comprehensive discussion of all these options and their respective merits and drawbacks would already suffice to fill a paper on its own. Given that our focus in this paper is a rather different one—viz., to show how a hybrid account of laws can be developed based on the DTA account—we cannot provide such an overarching discussion here. What we will therefore do instead is to merely put forward one way in which the problem can be addressed. This option draws on the discussion in Friend (2016), but has, to our knowledge, not yet been discussed in the context of the DTA account. We would like to stress, however, that this proposed solution is a purely optional element of our overall account, and that the reader should feel free to replace it by her preferred take on the form of the laws problem.

Based on a consideration of a variety of laws in physics and other sciences, Friend (2016) argues that all laws of nature are of a conditional form, and, more precisely, of the form $\forall x (Fx \rightarrow Gx)$. In this schema, the ‘*F*’ corresponds to what one may call a general condition that a physical/chemical/biological/...system of interest has to fulfil to be subject to the overall (higher-order) behavioural property expressed by functional relations between first-order properties (encoded by *G*). His basic argument in favour of this view is that functional laws on their own are just mathematical tautologies; they need to be made sense of by qualifying under which conditions they apply to which entities in the world, that is to which sort of system. This required qualification, however, automatically engenders that law statements are conditionals. (The only exception here would be given by functional relations that apply under all conditions to any kind of objects; Friend is quick to point out that current physics does not provide us with any sensible candidates for non-trivial functional laws of such a sort.)

Our proposal based on this argument is then straightforward: The properties related by the ${\mathcal {N}}$-relation correspond to the *F* and *G* in Friend’s account.^21^

To illustrate how Friend’s account works, let us return to our two main examples. Following Friend, Coulomb’s law may then be more explicitly taken to say that all two-charge systems will experience electrostatic components of force proportional to the product of each other’s charge according to the equation $F_Q= F_q = \frac{1}{4 \pi \epsilon _0} \frac{Q q}{r^2}$.^22^ Consequently, the proposed account maintains that this law is due to the *N*-relation holding between the property ${\mathcal {F}}$ of being a two-charge system on the one hand, and the property ${\mathcal {G}}$ of experiencing electrostatic components of force proportional to the product of each other’s charge according to the equation $F_Q= F_q = \frac{1}{4 \pi \epsilon _0} \frac{Q q}{r^2}$ on the other. Pauli’s exclusion principle says that all closed systems containing two fermions will be such that there is *some* difference in the quantum numbers of the two fermions. On our account, ${\mathcal {F}}$ would thus correspond to the property of being a closed system with two fermions, and the property ${\mathcal {G}}$ to the property of being such that there is *some* difference in the quantum numbers of the two fermions. In sum, the proposed account can accommodate laws of any given form, regardless of whether they involve functional relationships or not. And thus, the account evades the form of the law problem in a straightforward and fully general way.

The second of the two commonly mentioned problems for the DTA theory is the so-called *inference problem*—which is more directly relevant to our main concern in this paper, namely the modal status of the laws, than the form of the law objection. While the name traces back to van Fraassen (1989b), Lewis (1983) provides the iconic formulation of the problem for Armstrong’s account:

“Whatever N may be, I cannot see how it could be absolutely impossible to have N(F,G) and Fa without Ga. [...] The mystery is somewhat hidden by Armstrong’s terminology. He uses ‘necessitates’ as a name for the lawmaking universal N; and who would be surprised to hear that if F ‘necessitates’ G and a has F, then a must have G? But I say that N deserves the name of ‘necessitation’ only if, somehow, it really can enter into the requisite necessary connections. It can’t enter into them just by bearing a name, any more than one can have mighty biceps just by being called ‘Armstrong’.” (p. 366)

The question raised here is this: Why does ${\mathcal {F}}$’s and ${\mathcal {G}}$’s standing in the ${\mathcal {N}}$-relation entail, i.e. necessarily imply, that all *F*s are *G*s? The answers that have been suggested in the literature fall into broadly two camps.

The first variety of answers, which may be labelled ‘heavyweight solutions’, seek to explain the entailment by substantive metaphysical theorising concerning the nature of universals and states of affairs (Armstrong 1983, 1997; Tooley 1987 (labelled Tooley’s ‘speculative solution’)). These proposed solutions, however, are commonly taken to be affected by severe problems and ultimately unpromising.^23^ ‘Lightweight solutions’, on the other hand, account for the entailment simply by maintaining that it forms part of the DTA theory’s very own theoretical posits, i.e., the axioms stipulated by the theory. While Tooley (1987) himself proposes such a solution (the ‘solution by stipulation’) as well, he sees it as unsatisfactory on its own, and thus in need of being amended by the speculative solution. Schaffer (2016), by contrast, argues that the lightweight solution is already sufficient: According to him, the proponent of the DTA account need not do more than to invoke (Necessary Implication) as an axiom of her overall theory, full stop.^24^

Drawing on this distinction between heavyweight and lightweight solutions, we might conceive of the crucial question underlying the *inference problem* as follows: Is there a metaphysical explanation for the entailment and if so, what is it? Then, the inference problem itself might be seen as the following worry: The claim that $N({\mathcal {F}}, {\mathcal {G}})$ entails that all *F*s are *G*s is not a good candidate for a metaphysical stopping point and there is no good candidate for something that metaphysically explains it either—due to additional, specific commitments of the DTA theorists, or maybe even for fully general reasons. Seeing the dialectics in this way, it seems that merely pointing out that this claim is an axiom of the DTA theory does not help much to dispel the worry behind the inference problem. While it might indeed be considered a legitimate move to invoke this claim as an axiom rather than to offer an explanation of it, the specific worry would still stand. Moreover, taking a stance regarding the question of whether this claim is a brute truth or has a metaphysical explanation in other terms seems generally preferable in order to have a ‘full-blown’ account of the laws of nature. Thus, when developing our own version of the DTA theory in the following section, we also take up the task of offering an answer to the inference problem.

### The essentialist DTA account

As we have seen, the fact that all laws turn out to be contingent on the DTA account is due to the fact that whether properties stand in ${\mathcal {N}}$-relations is contingent, plus potentially the idea that $N({\mathcal {F}},{\mathcal {G}})$ merely implies but does not entail (i.e. necessarily imply) that all *F*s are *G*s. Our proposal about how to make room for both metaphysically contingent and necessary laws within the DTA account is then quite simple: We propose to maintain first that $N({\mathcal {F}},{\mathcal {G}})$ entails that all *F*’s are *G*’s, and, second, that merely *some* universals stand contingently in ${\mathcal {N}}$-relation, while *others* do so necessarily. That is, our account replaces (Contingent Holding) with the following thesis:

- Mixed Holding: $$  \exists {\mathcal {X}}_{1},{\mathcal {Y}}_{1} ( N({\mathcal {X}}_{1}, {\mathcal {Y}}_{1}) \; \& \; \diamond \lnot N({\mathcal {X}}_{1}, {\mathcal {Y}}_{1})) \; \& \; \exists {\mathcal {X}}_{2}, {\mathcal {Y}}_{2} \; \Box N({\mathcal {X}}_{2}, {\mathcal {Y}}_{2})$$.

In words: There are some properties which stand in the ${\mathcal {N}}$-relation, but which do so only (metaphysically) contingently, and there are others which necessarily stand in the ${\mathcal {N}}$-relation. Given (Necessary Implication), lawlike generalizations that are due to a contingently holding ${\mathcal {N}}$-relation thus turn out to be contingent, while laws that are due to a necessarily holding ${\mathcal {N}}$-relation turn out to be necessary. In this way, the account naturally gives rise to a non-absolutist view of the modal status of laws. In our two main example cases, for instance, the relevant properties ${\mathcal {F}}$ and ${\mathcal {G}}$ would be taken to stand necessarily in ${\mathcal {N}}$-relations in the case of the PEP, and merely contingently in the case of Coulomb’s law. And thus, the PEP turns out to be necessary, while Coulomb’s law turns out to be contingent, just as they should.

There is now, however, the further question of what the sources of the necessities in (Mixed Holding)—and prior to this—in (Necessary Implication)—are. Our preferred answer to both is in terms of *essence*.

More specifically, in the case of (Necessary Implication), we propose that necessity is due to *the essence of the* ${\mathcal {N}}$-*relation*.^25^ Grounding the necessity in (Necessary Implication) in the essence of the relevant ${\mathcal {N}}$-relation is to say that it is part of the very nature of ${\mathcal {N}}$ that it gives rise to the corresponding regularities. Letting the operator ‘$\Box _{xx} \phi$’ stand for ‘it is essential to *xx* that $\phi$’, where *xx* may range over objects, properties, or a plurality of objects, a plurality of properties, or of both, we thus have:

- Essence of $${\mathcal {N}}$$: $$\Box _{{\mathcal {N}}} (N({\mathcal {F}}, {\mathcal {G}}) \rightarrow \forall x (Fx \rightarrow Gx))$$.

In words: It is essential to ${\mathcal {N}}$ that, if ${\mathcal {F}}$ stands in the relation ${\mathcal {N}}$ to ${\mathcal {G}}$, then everything which is *F* is *G*.

Moreover, we combine this essentialist claim with the (by now well-established) view that essence is not to be analysed in modal terms, but that it is rather essence which is a source of modality.^26^ In particular, according to the resulting account, $$\Box _{xx} \phi $$ metaphysically explains $$\Box \phi $$ for any *xx* and $$\phi $$, since it specifies the source of the metaphysical necessity of the latter claim. In this way, (Essence of $${\mathcal {N}}$$) yields a straightforward explanation of why (Necessary Implication) holds, thus providing an answer to the inference problem.^27^$$^{,}$$^28^

In the case of (Mixed Holding), we also wish to propose that the notion of necessity at play is grounded in essence. Here, the idea is that some properties stand essentially in *N*-relations, while others do so merely accidentally:

- Mixed Essential Holding: $$  \exists {\mathcal {X}}_{1}, {\mathcal {Y}}_{1} ( N({\mathcal {X}}_{1},{\mathcal {Y}}_{1}) \; \& \; \lnot \exists xx \Box _{xx} N({\mathcal {X}}_{1},{\mathcal {Y}}_{1})) \& \exists {\mathcal {X}}_{2}, {\mathcal {Y}}_{2} \; \Box _{{\mathcal {X}}_{2}, {\mathcal {Y}}_{2}} N({\mathcal {X}}_{2},{\mathcal {Y}}_{2})$$.^29^$$^{,\text { }}$$^30^

In words: There are some properties which stand in the ${\mathcal {N}}$-relation, but whose standing in that relation is not essential to anything, and there are others which essentially stand in the *N*-relation. (Mixed Essential Holding) provides us with a straightforward, and, we think, very natural explanation of (Mixed Holding): The former part of the condition—viz., $\exists {\mathcal {X}}_{1}, {\mathcal {Y}}_{1} (N({\mathcal {X}}_{1},{\mathcal {Y}}_{1}) \; \& \; \lnot \exists xx \Box _{xx} N({\mathcal {X}}_{1},{\mathcal {Y}}_{1}))$—provides for the contingency of some properties’ standing in the ${\mathcal {N}}$-relation, while the latter part—viz., $\exists {\mathcal {X}}_{2}, {\mathcal {Y}}_{2} \; ( \Box _{{\mathcal {X}}_{2}, {\mathcal {Y}}_{2}} N({\mathcal {X}}_{2},{\mathcal {Y}}_{2}))$—provides for there being properties which stand essentially, and thus metaphysically necessarily, in them. Coming back to our two main example cases, the account would thus maintain that, in the case of the PEP, the relevant properties ${\mathcal {F}}$ and ${\mathcal {G}}$ stand essentially in the ${\mathcal {N}}$-relation. In the case of Coulomb’s law, by contrast, we would have that, even though the relevant properties ${\mathcal {F}}$ and ${\mathcal {G}}$ stand in the ${\mathcal {N}}$-relation, it is not part of the essence of them, or any other plurality of entities, that they do so.

Now, our account, as it stands, leaves it open whether, in the case of the PEP, the essence is ultimately a joint essence of both ${\mathcal {F}}$ and ${\mathcal {G}}$, or merely one of the two. A closer look at the properties involved, however, makes it plausible that the ultimate essence bearer is ${\mathcal {F}}$ alone if our solution to the form of the laws problem is assumed. To see this, recall that, following Friend, we take ${\mathcal {F}}$ to be a property that picks out the relevant sort of system—in the case of the PEP, the property of being a two-fermion system—and ${\mathcal {G}}$ a property that corresponds to what the law says about systems of this sort—for the PEP, the property of being such that the fermions do not have the same quantum numbers. In this case, it seems very plausible that it is already essential to ${\mathcal {F}}$ alone that it stand in ${\mathcal {N}}$ to ${\mathcal {G}}$. Moreover, we will also see in Sect. 3 that a careful investigation of our physical theories speaks in favour of this conclusion. That being said, however, we want to keep our overall account neutral with regard to the question of whether we should adopt this specific solution to the form of the laws problems and thus conceive of ${\mathcal {F}}$ and ${\mathcal {G}}$ in this way. And therefore, we formulate (Mixed Essential Holding) in a way that is neutral with regard to whether ${\mathcal {F}}$, ${\mathcal {G}}$ or both taken together the ultimate bearers of the essence.

It is worth noting that in endorsing the essentialist line of response to the inference problem, we have to eschew Armstrong’s quidditist account of properties.^31^ Note, however, that the commitment to quidditism is by no means an integral part of the DTA account as such, but rather a separate commitment which Armstrong takes up for independent reasons. Hence, quidditism is a purely optional element for the proponent of the DTA account, and endorsing the DTA account should not by itself compel us to be sceptical about essence.

One might object to the proposed account that, even when constructing a non-absolutist view of laws, as soon as we help ourselves to essence, adopting the nomic necessitation account in addition would look like a bad idea for reasons of ideological parsimony: A purely essentialist account would seem to be sparser than an account that invokes both essence and the ${\mathcal {N}}$-relation, and thus, other things being equal—and, in particular, if it could also account for different modal statuses of laws—preferable. But this is, so far, wishful thinking: As we have seen in Sect. 1.3, the two essentialist non-absolutist views of laws proposed thus far either fail to meet the three explanatory challenges for such accounts or need to rely on a contentious account of properties. Hence, in the absence of a purely essentialist non-absolutist account which proves otherwise, other things are not equal, and the criterion of parsimony does not enter the picture. Rejecting the proposed account on the basis of parsimony considerations would thus be misguided.

Finally, it is worth stressing that, beyond the essentialist understanding of metaphysical necessity, various alternative accounts would be available—e.g. reductionist accounts based on possible worlds (Lewis 1986); counterfactual accounts (Williamson 2007; Lange (2009)), non-reductionist views (Forbes 1989; Wang 2013; Wilsch 2017); and conventionalist views (Sidelle 1989; Sider 2011). A foe of essence could thus instead invoke one of these accounts in order to explain the necessities in (Necessary Implication) and (Mixed Holding). Moreover, we do think that it would also in principle be viable to simply take the relevant necessities to be brute necessities. While such a move arguably stands in tension with further commitments of DTA—such as, Hume’s dictum (i.e., the ban on necessary connections among distinct entities) and the thesis that only analytic truths can be necessary—these further commitments do not form an integral part of the DTA view as such, and are rather further optional commitments. (See Hildebrand 2013 for discussion.)

That being said, however, it should be clear that there are advantages for not taking the necessities to be brute. First, by instead providing an (essentialist) explanation for the necessity in (Necessary Implication), one is able to provide an answer to the inference problem. Second, there are considerations that tell in favour of invoking essence to account for the necessity in (Mixed Holding): As we will argue in Sect. 3, a careful investigation of physical theories suggests that metaphysically necessary laws indeed express essential features of the properties that are ${\mathcal {N}}$-related. Thus, while we take essence to be an in principle optional element of the account as presented in this section, we think that there are good reasons to endorse it.

### How the essentialist DTA account meets the explanatory challenges for non-absolutist theories

Let us now see how our proposed amended DTA view can meet the challenges set out in Sect. 1.2. First, the proposed view can easily avoid the synchronization problem. Nomic necessity, on our view, is naturally understood as a conditional, or relativized form of metaphysical necessity: metaphysical necessity *given* all the actual instances of the ${\mathcal {N}}$-relation holding between universals. More precisely, a nomically necessary proposition *p* is one that follows, as a matter of metaphysical necessity, from the proposition, $\varPhi$, which says that ${\mathcal {N}}$ holds between ${\mathcal {F}}_1$ and ${\mathcal {G}}_1$, ${\mathcal {N}}$ holds between ${\mathcal {F}}_2$ and ${\mathcal {G}}_2$, and so on, where the (${\mathcal {F}}_i, {\mathcal {G}}_i$)s are all the pairs of universals related by ${\mathcal {N}}$. Using ‘$\Box$’ as the sentential operator expressing metaphysical necessity and ‘$\boxminus$’ to express nomic necessity, we can thus define:

- Definition: nomic necessity $\boxminus p := \Box (\varPhi \rightarrow p)$^32^

In words: *p* expresses a nomic necessity if, and only if, (per definition) it is metaphysically necessary that if the actual ${\mathcal {N}}$-relations hold, then *p*. It clearly follows that the metaphysical necessities form a subset of the nomic necessities, so that, trivially, any metaphysically necessary law will also be a nomically necessary law. As a result, our proposed non-absolutist view of the modal status of the laws easily avoids the synchronization problem.

Second, and relatedly, our view meets the challenge of explaining, not only why both sorts of laws are *nomically* necessary but also why some laws are metaphysically necessary and others metaphysically contingent (double explanatory challenge). Both sorts of laws are nomically necessary because both are metaphysically entailed by the holding of the ${\mathcal {N}}$-relation between the universals that are actually ${\mathcal {N}}$-related—proposition $\varPhi$ above. What distinguishes the metaphysically necessary laws from the metaphysically contingent ones is that the former, unlike the latter, are *also* metaphysically entailed by the empty set of propositions. And this, in turn, is naturally explained by the fact that, in the case of metaphysically necessary laws, but not in the case of metaphysically contingent laws, the ${\mathcal {N}}$-relation between the universals involved holds as a matter of metaphysical necessity (because it holds in virtue of the essence of these universals).

Third, our proposed view provides us with a criterion to distinguish *all* laws of nature, whatever their modal status, from the other true universal generalizations (common lawhood criterion challenge): A law of nature, independent of its modal status, is a universal generalization of the form ‘All *F*s are *G*s’ such that the universals involved, ${\mathcal {F}}$ and ${\mathcal {G}}$, are ${\mathcal {N}}$-related. By contrast, the universals involved in an accidentally true generalization which is not a law are not ${\mathcal {N}}$-related. For instance, the proposition that all electrons have negative charge is putatively a true universal generalization, indeed a law of nature,^33^ because the two properties involved are ${\mathcal {N}}$-related. By contrast, the proposition that all gold spheres are less than 17 kilometres in diameter may be a true universal generalization, but as the property of being a gold sphere and the property of being less than 17 kilometres in diameter fail to be ${\mathcal {N}}$-related, it is not a law of nature. Thus, our account offers a clear criterion for lawhood which is neither solely dependent on the modal status of the laws, nor merely disjunctive.

## Linking the essentialist DTA account back to physics

The essentialist DTA account provides a metaphysical framework that explains how laws can have different modal status. In the previous sections, we have argued that this account does not suffer from the problems which affect previously proposed non-absolutist accounts. Remember, however, that the main motivation for introducing a non-absolutist account of the laws of nature is naturalistic: As stressed in both Tahko (2015) and Hendry and Rowbottom (2009), science gives us reasons to reject the absolutist view of the modal status of the laws of nature forced upon us by standard theories of the laws like dispositional essentialism. A central question that remains to be addressed is thus: Is the essentialist DTA account in line with this naturalist approach? While giving a wholly general answer to this question (i.e., an answer which takes into account potential laws of nature from all relevant scientific disciplines) is beyond the scope of this paper, we will give a partial answer by focusing on the laws of physics.

With respect to physics, the main claim of non-absolutist theories such as the essentialist DTA account is that there is a two-fold distinction between those laws of physics which are merely nomically necessary and those which are metaphysically necessary. This means that a non-absolutist account can, given our restricted focus, only count as adequate from a naturalistic point of view if our best physical theories indeed suggest that physical laws are necessary in these two distinct senses. In this section, we will make two interrelated points then: First, that the kinematical/dynamical distinction—a distinction that lies at the heart of physical theorising—directly aligns with a two-fold distinction between laws which are necessary in a weaker and in a distinct stricter sense. Second, that the kinematical/dynamical distinction at least strongly suggests that the stricter kind of necessity is metaphysical necessity.^34^ Before, we want to quickly address two in-principle worries about referring to metaphysical necessity in the context of physical theories.

### Physical laws and metaphysical necessity

How in general can it happen that certain statements of a physical theory can express metaphysical, rather than merely nomic necessities?^35^ Proponents of a non-absolutist view have to provide a satisfying answer to this question and, as the question insinuates, they cannot do so. Physical theories do not explicitly talk about modality, so this worry must be founded on an underlying philosophical view. The view in question has it that metaphysical necessity is absolute, in the sense that a proposition *p* can only count as expressing a metaphysical necessity if its negation expresses an impossibility *in any sense of ‘impossible’*. One may after all argue that laws of physics can never count as metaphysically necessary, since it is easy to find a sense in which their negation expresses a possibility, e.g. because it is logically consistent or conceivable in some possibility-conducive sense. Notably, a view of this sort, which is crucially based on the idea that the notion of possibility is constrained by a liberal principle of recombination, was proposed by Lewis (see Lewis 1986, section 1.8.) and also provided the basis for Armstrong’s theory of modality (Armstrong 1989). It shouldn’t come as a surprise that the essentialist DTA account is based on a rejection of this view of metaphysical modality.^36^ Its essentialist component places restrictions on the recombinability of the building blocks of reality, including in particular those imposed by the essential relations between properties linked by metaphysically necessary laws. Such relations neither hold as a matter of logic, nor can they be established on a purely a priori-basis. Accordingly, we, like many other philosophers who work with a substantive notion of metaphysical modality (cf. Gendler and Hawthorne 2002), deny that the logical consistency or conceivability of a state of affairs is sufficient for its metaphysical possibility and hence reject the view in question.

The second and related worry concerns the fact that actual laws in physics are always posited as part of physical theories. Theories have specific ranges of applications, e.g. applying at a particular scale, and they are, at least in principle, revisable. But given that metaphysical necessities are supposed to express theory-independent and unrevisable truths, how then could an actual physical law ever attain this status?

Let us first point out that the notion of metaphysical modality was from the very start explicitly introduced as an objective, worldly notion in opposition to views of modality which equated it with a priority or analyticity. In the book which did the most to re-establish a metaphysical notion of modality, *Naming and Necessity*, Kripke explicitly embraces the idea that certain physical statements express metaphysical necessities.^37^ According to the classic Kripkean view, when making de re metaphysical modal claims about a natural kind, such as e.g. electron, we do not refer to some possible kind which resembles, or in some other way corresponds to the actual natural kind. Rather, we make those very same kinds part of the possible worlds relevant to our modal claims by stipulation. One way to spell out the implications of this view for the laws of nature has it that laws of nature are metaphysically necessary, because they capture the essences of natural kinds. These essences have modal import in the sense that they constrain the space of metaphysical possibilities by ruling out those possibilities which fail to conserve the actual essential properties of those natural kinds.

While we do, of course, not accept this particular approach to the necessity of the laws, which after all amounts to a version of dispositional essentialism, we accept the underlying idea that it is part of the very idea of metaphysical necessity that (certain) physical laws can in principle capture essential traits of the actual world, or entities in it, which serve as constraints on the extent of what is and what is not metaphysically possible. We in particular align ourselves with the broadly Kripkean, essentialist view of metaphysical modality developed by Fine, Lowe, Correia and others which we worked with in the previous section.^38^ But the question re-arises: Are we justified to assume that the *laws of current physical theories* capture such essential traits?

There are different ways to respond to this question. First, one might concede that there is currently no such justification, but assert that the laws of physics which earn the status of metaphysical necessities are laws of an ideal, completed theory.^39^ The underlying move of referring to a final, completed physics is a standard move made by many naturalistically minded philosophers. This response is conservative from a metaphysical perspective, since it respects the mentioned standard assumptions about metaphysical modality and the corresponding views about essence. Its drawback is that it entails that all concrete discussions which treat current physical laws as laws of nature are merely tentative and potentially subject to later revision.

A second, less metaphysically conservative response is to adopt a non-standard, relativized notion of metaphysical necessity which affords this status to theory-relative and potentially revisable claims like the current laws of physics. Some steps towards a view of this sort have been taken in the literature (see Murray and Wilson 2012), but further work for which this paper is not the right place is needed to flesh out this response. A naturalistically acceptable non-absolutist view may incorporate either of these responses.^40^ While we have confined ourselves to considerations of a more general sort in this subsection, a more specific discussion in light of the kinematical/dynamical distinction will follow later in this section.

### The kinematical/dynamical distinction introduced

As we will argue in the remainder of Sect. 3, at a theory-relative level, the distinction between *kinematical vs. dynamical* structure à la (Curiel 2011, 2014, 2016) does not only suggest the adequateness of a non-absolutist account; it can at the same time also serve as a criterion for which laws can count as metaphysically necessary (they are part of the kinematical structure of the theory), and which laws can at most count as nomically necessary (they are part of the dynamical structure of the theory).

Generally, Curiel characterises the kinematical/dynamical distinction as the distinction between, on the one hand, mere states of a system (which can involve both time-independent and time-dependent quantities) as well as their constraints (called ‘kinematical constraints’) and, on the other hand, context-dependent restrictions (including possible evolution) of such states (over and above the kinematical constraints, referred to as dynamics).^41^ More precisely, kinematical constraints as opposed to dynamical structure refer to *concrete* relations in terms of the basic kinematical variables that are invariant across all possible models of the theory (cf. Curiel 2016). The term ‘concrete relation’ contrasts with ‘placeholder relation’, that is, a relation that features quantities that still need to be fixed in terms of the basic kinematical variables. In a 1-dim. theory of Newtonian mechanics with basic variables *x* and *v*, ${\dot{x}} = v$ is a concrete relation, and the force law $F = m \ddot{x}$ a placeholder relation; the concrete form of *F* still needs to be fixed further in terms of the basic variables *x*, *v* and certain constants—as for instance done by setting $F := \frac{G m_1 m_2}{x^2}$ in certain scenarios—cf. Curiel 2016, p. 5). (Clearly, a non-trivial placeholder relation can thus only be dynamical but not a kinematical structure as it will not be the same for all models to begin with.)

A good illustrative example of the distinction in use is in the context of Maxwell electrodynamics.^42^ The two homogeneous Maxwell equations

$$\begin{aligned}&\nabla \cdot {\mathbf {B}} =0\\&\nabla \times {\mathbf {E}} +{\frac{\partial {\mathbf {B}} }{\partial t}}=0\\ \end{aligned}$$

are the same for any model of Maxwell electrodynamics when expressed in terms of basic dependent variables ${\mathbf {E}}$ and ${\mathbf {B}}$ and independent variables *x*, *y*, *z*, *t*. In contrast, the other two Maxwell equations (dubbed ‘inhomogeneous’)

$$\begin{aligned} \nabla \cdot {\mathbf {E}}&={\frac{\rho }{\varepsilon _{0}}}\\ \nabla \times {\mathbf {B}} -{\frac{1}{c^{2}}}{\frac{\partial {\mathbf {E}} }{\partial t}}&=\mu _{0}{\mathbf {J}} \end{aligned}$$

differ from model to model depending on which external charge density $\rho$ and current ${\mathbf {J}}$ are chosen as sources. Thus, contra common presentation, two of the four Maxwell equations are in fact kinematical and not dynamical according to the distinction as drawn by Curiel.

At this point, it is important to stress that the notion of kinematics has to be distinguished from the notion of ‘kinematics’ qua subdiscipline as common in the context of (classical) mechanics. The sense in which Curiel (and we) use the term ‘kinematics’ is in terms of the kinematical structure of a physical theory. This is arguably also the standard usage of the term outside of classical mechanics, say in quantum mechanics and GR.^43^ In our eyes, Curiel can take credit for providing the only explicit account of the distinction despite its *omnipresence* in the practice of physical theorising (see next subsection).

How to think of kinematical constraints over and above their *characterisation* through form invariance? Kinematical constraints are statements which are needed to minimally pick out the systems of interest to be described by the theory *before* and *in virtue of which* dynamical evolutions can be set up. In other words, kinematical constraints are necessary conditions for dynamical equations—in fact, for the theory as a whole—to be about something. This can be understood merely semantically, or, and that’s what we are after, ontologically.

We claim that (i) the kinematical/dynamical distinction à la Curiel is omnipresent in our best physical theories (see the next subsection and the appendix; we can of course not provide fully detailed case studies for all relevant physical theories here but just a caleidoscopic overview). Secondly, we claim that (ii) the kinematical/dynamical distinction à la Curiel can naturally be taken to motivate a non-absolutist account of the modal status of physical laws, if one is open to accept an account of the modal status of laws in the first place. In light of (ii) then, our attempt to find an attractive non-absolutist account of laws is promoted from a rich conceptual exercise to a stab at the very nature of reality.

No matter whether we are interested in reading out metaphysics from our best physical theories, or probing the adequateness and usefulness of certain metaphysical concepts in the face of them—we need to be clear at each relevant length/energy scale which theories are our *best* physical theories:^44^ Although from a textbook perspective the choice of a best theory (interpretation) seems unambiguous at many scales, surely the situation is not that simple at a second glance. Newtonian mechanics, for instance, has several formal representations partly different in scope of application (Newtonian force representation; Lagrangian representation; Hamiltonian representation; and even a spacetime representation) that in many cases should go along with different forms of theory interpretation. Needless to say, the issue of underdetermination does not improve when moving towards theories of the quantum realm.

Nevertheless, we can always try to make observations on what *all* these different theories and their interpretations have in common and meta-inductively infer that future physical theories display these commonalities as well. And indeed, such an observation is suggesting itself with respect to what is generally called the kinematical/dynamical distinction: The current omnipresence of a kinematical/dynamical distinction across theories (as indeed prompted through claim (i) once established) motivates the assumption of a kinematical/dynamical distinction with respect to our future best physical theories yet to come, in turn suggesting modal pluralism among the laws of physics (provided claim (ii) holds).

### Kinematical constraints across physics

The kinematical/dynamical distinction in the sense of Curiel is relevant for all physical theories that deal with physical processes, i.e. classical theories, (general) relativistic theories, and quantum mechanical theories. For instance, in general relativity, spacetime structure is usually seen to become (partly) dynamical—as opposed to kinematical in special relativity; in quantum gravity, one talks about solving kinematical as opposed to dynamical constraints, etc. To make this point explicitly, we have compiled several instances of kinematical constraints across the board of various spacetime theories, including a quantum gravitational one, in the appendix. Although not a complete list, the choice is in so far representative as it includes instances of non-relativistic, relativistic, and quantum physics. Even if the reader may not wish to consult the admittedly quite technical appendix and trust us in our judgement here, it is important to appreciate the following specific lesson from it: what one calls ‘kinematical’ and ‘dynamical’ depends on the theory-interpretation at stake, and cannot be merely based on joined formal structure shared across different interpretations of one and the same ‘theory’.

Our analysis in the appendix grounds another significant observation: By noting the unbroken presence of the distinction *throughout* our past and current candidates for best physical theories, one can defend the claim that the specific extrapolation from available physics to future physics is much more stable than a naive meta-induction from the metaphysics of our *currently* best physical theories alone as to what the putative metaphysics of an (expected) final theory could be. This is an important point as in particular McKenzie (2019) has argued against the reliability (if not even possibility) of metaphysical extrapolations from current physics to a (fundamental) metaphysical structure.

More precisely, McKenzie has the concern that metaphysical statements are not statements prone to approximation but rather yes-or-no-affairs (think of structuralism as the idea that there are no intrinsic properties, for instance) whose answers can putatively wildly oscillate across theory changes. Although we beg to differ on McKenzie’s assessment more generally, in the context of the current work all we need to convince the reader of is that kinematical constraints as a category (just like dynamical laws as a category) have been present, are present and are likely to be present in our best future theories, and that this sort of meta-induction then rests on acceptable footing. That this footing is solid, seems to follow from the fact that (1) our extrapolation rests on taking into account a whole convergence trend analogously to how extrapolations work in science, and that (2) we usually accept extrapolations in science of this kind too.^45^

### Kinematical constraints, modal non-absolutism, and metaphysically necessary laws

A theory’s kinematical constraints minimally—and in some sense thus definitionally—characterise the systems it is supposed to model. They thereby form the basis of any modelling of putative dynamical evolutions in the first place. Curiel (2011) puts it as follows:

[physical] frameworks do not predict kinematical constraints; they demand them. I take a prediction to be something that a theory, while meaningfully and appropriately modelling a given system, can still get wrong. Newtonian mechanics, then, does not predict that the kinematical velocity of a Newtonian body equal the temporal rate of change of its position; rather it requires it as a precondition for its own applicability. It can’t “get it wrong”. If the kinematical constraints demanded by a theory do not hold for a family of phenomena, that theory cannot treat it, for the system is of a type beyond the theory’s scope. (p. 12)

There is a quite natural way to understand the distinction between kinematical and dynamical constraints in modal terms: Kinematical constraints give rise to a stronger sort of necessity—or equivalently, constrain a broader space of possibilities. There is a sense in which a physical system *must* evolve in the way prescribed by dynamical laws; but the sense in which that system *must* conform to the relevant kinematical constraints is a stronger one. Not only is the set of worlds in which the dynamical laws hold a proper subset of the set of worlds in which the kinematical laws hold. But more than that: The existence of a kinematic/dynamical *distinction* strongly suggests that laws of nature may have two qualitatively distinct modal statuses.^46^

How does this relate to our previous discussion of metaphysical accounts of laws and their modal status? First of all, it gives an advantage to the non-absolutist accounts of laws discussed in Sects. 1 and 2, such as our proposed essentialist DTA view, over absolutist accounts: Unlike the former, the latter—on which all laws have the same modal status (be it metaphysical necessity or mere nomic necessity)—are simply not compatible with the modal divide suggested by the kinematical/dynamical distinction. This is already an important point, given the popularity that absolutist views still enjoy in metaphysical debates about laws. Moreover, the kinematical/dynamical distinction provides non-absolutists with a naturalistic criterion to determine, in practise, which law has which modal status.

For instance, relying on that distinction, the non-absolutist can give naturalistic support—rather than the mostly intuition-based support it e.g. receives in Tahko (2015)—to her claim that Coulomb’s law is necessary in a strictly weaker sense than the Pauli Exclusion Principle (PEP): the former is a dynamical, the latter is a kinematical law. While it should be clear that Coulomb’s law is a dynamical law, the case of the PEP is a bit more intricate. To realise this, first, recall that the PEP states that “no two fermions in a closed system can occupy the same quantum state at the same time” (Tahko 2015, p. 514). Its generalisation—the spin-statistics connection—says that (1) fermions obey Bose-Einstein statistics, and (2) bosons obey Dirac-Fermi statistics; the fact that fermions obey Bose-Einstein statistics is a technical statement that importantly implies that no two fermions can have the same state. Now, in non-relativistic quantum mechanics, the Pauli-Exclusion Principle and the spin-statistics connection more generally are brute principles; in this context, we can only state that the principle is kinematical in so far as that it is supposed to hold in all models qua brute principle. However, once considered in the richer framework of quantum field theory, the spin-statistics connection becomes a provable theorem. Interestingly, no single generally accepted formal framework of QFT exists as of now. What can be said nevertheless then is that these proofs of the spin-statistics theorems work with four basic types of premises, namely (i) some form of Lorentz invariance of the involved fields, (ii) the spectrum of the Hamiltonian, (iii) causality, (iv) finite particle multiplicity, and—arguably of even more technical nature—(v) analyticity (see Swanson 2018).^47^ Evidently, the spin-statistics connection—including the Pauli exclusion principle as a corollary thereof—can be identified as a kinematical statement if the premises of the proof are of kinematical nature. This seems to be the case though: Notably, the requirement of Lorentz-invariance here is just the general posit that field theoretical objects are covariant/invariant under Lorentz-transformations; all other premises are un-problematically kinematical in nature to begin with. In consequence then, the spin-statistics theorem and thus the Pauli-Exclusion Principle again have to be understood as kinematical.^48^

Up to now, we have seen that the kinematical/dynamical distinction tips the balance in favour of non-absolutism over absolutism, and that it provides the former kind of theory with a clear criterion to determine which laws have the stronger and which laws have the weaker modal status. However, the non-absolutist views considered earlier, in particular our own proposed view, do not only claim that there are two modal statuses for laws—they make the further claim that the stronger modal status is *metaphysical necessity*. And this further claim is not obviously supported by the kinematical/dynamical distinction itself.

That the only two modal statuses at issue are those of nomic and of metaphysical necessity is a shared assumption in metaphysical debates about laws of nature and in particular in the debate this paper contributes to. *According to that assumption*, the modal divide suggested by the kinematical/dynamical distinction in physics does directly lend support to the sort of non-absolutist view considered previously, on which the modally stronger laws are specifically *metaphysically* necessary. But does this assumption itself also find some naturalistic support in the kinematical/dynamical distinction? Is there reason to think that kinematical laws are best understood as being metaphysically necessary, especially in the essentialist sense assumed in our previous discussion—as opposed to having, say, just a higher “grade” of nomic necessity than dynamical laws?^49^

Even if not the only possible one, a reading of kinematical laws as metaphysically necessary, as essential to the physical entities to which they apply, seems quite plausible. Indeed, as the passage quoted above from Curiel (2016) may already suggest, his discussion of kinematical constraints lends itself to such a reading.^50^ According to him, one main role that kinematical constraints, unlike the dynamics, play in physical theory is that ‘they characterise the physical *nature* of systems the theory treats, i.e., [they are] *constitutive* of the kind of system the theory treats’ (Curiel 2016, p. 9; emphasis added). The notions of nature and constitutiveness at play here may be interpreted in a purely semantical way—in terms of the meanings of the physical terms involved (this is actually the view which Curiel (2016) ultimately favours). However, his discussion also leaves room for a more *ontological* interpretation, which indeed suggests itself quite naturally: We may say that the kinematical constraints capture those aspects of a system which make it the sort of physical system it is according to the theory, paralleling almost exactly the Aristotelian characterisation of essence as the ‘what it is to be’, or the ‘real definition’ of an entity.^51^ Kinematical constraints in a physical theory hence play exactly the same role which essential characteristics of entities play in metaphysical theorising. Given the standard assumption that essentiality entails metaphysical necessity (cf. Fine 1994), this systematic parallel strongly suggests that kinematical constraints are metaphysically necessary. Note, moreover, that the idea that kinematical aspects are essential to *systems* is compatible with our proposed take on the ‘forms of the law problem’ in Sect. 2.1: remember that we suggested that the relevant ${\mathcal {N}}$-relation holds between some property ${\mathcal {F}}$ which singles out the relevant type of system, and some property ${\mathcal {G}}$ which captures the behaviour of this system through a functional relation of its first-order properties. This behaviour can, after all, still be constitutive—if so, the law as a whole becomes metaphysically necessary. For example, in the case of the putatively metaphysically necessary PEP, the account offered in Sect. 2.1 maintains that ${\mathcal {F}}$ corresponds to the property of being a closed system with two fermions, and ${\mathcal {G}}$ to the property of being such that there is some difference in the quantum numbers of the two fermions.

Let us be clear about what our argument here is. Our starting point is that there is an explanatory need which can be met by an adequate non-absolutist theory of the laws of nature. We propose an ontological reading of Curiel’s characterization of the kinematical/dynamical distinction, according to which kinematical laws express essential truths. We have made it plausible that this is a viable reading, but also pointed out that it is not the only one available. We have also argued that *if* this reading is adopted, *then* kinematical laws are metaphysically necessary (by the implication from essence to metaphysical necessity). This together with the points made earlier in the paper gives us an *abductive* argument for our proposed reading: There is a need for an non-absolutist theory of the laws of nature. Such a theory has to meet certain constraints. We have introduced three such constraints in Sect. 1.2 and then argued in Sect. 2.3 that the essentialist DTA theory meets them. But to bolster our argument, two further constraints should be mentioned.

First, making an earlier requirement more explicit, the theory should be naturalistic, in the sense that it if informed by science to a sufficient degree, and second, it should furthermore provide us with a workable criterion for distinguishing the metaphysically necessary from the metaphysically contingent laws of nature. Regarding the latter constraint, we have recently argued that the criterion provided by Tahko is inadequate, which means that non-absolutists need to look elsewhere. (See Hirèche et al. 2021.) The essentialist DTA theory by itself does not meet the two further requirements, but it can do so if combined with the proposed criterion for the metaphysical necessity of the laws based on the kinematical/dynamical distinction: the theory on its own gives us a metaphysical explanation of *why* some laws are metaphysically necessary (because they hold in virtue of the nature/essence of some universals) and the ontic reading of the kinematical/dynamical distinction adds a naturalistic and workable criterion for the metaphysical necessity of laws which allows us to determine *which* laws have this special modal status. It should thus be clear that our argument for the ontic reading of the distinction is abductive; in the given dialectical context it provides a crucial part of the best available explanation, i.e. of an adequate non-absolutist theory of the laws of nature which both explains why some laws are metaphysically necessary and also allows us to determine which those laws are.^52^

Coming back to a point which has been raised at the beginning of this section, there remains one property of kinematical constraints which appears to undermine our claim that the kinematical laws are metaphysically necessary, namely their theory-relativity. After all, like metaphysical necessity, essence is a theory-independent notion: The essence of an entity consists in what it is to be that entity, and this is, essentialists believe, not determined by any theory about the entity, but rather an objective fact which obtains independently of what any theory, physical or of any other kind, says. Does this standard assumption about essence then threaten the two-fold thesis developed in this subsection, the thesis that physical laws which express kinematical constraints express essential truths and therefore metaphysical necessities?

That a kinematical constraint is theory-relative may mean different things, which may have different implications for our thesis. Kinematical constraints play a constitutive role concerning the type of system a physical theory is about, which gives us a first, wholly unproblematic sense in which they are theory-relative: Kinematical constraints are *specific* to the theory (and interpretation) to which they belong, which is of course compatible with them being absolutely, essentially, and metaphysically necessarily true. Theory-specificity in this sense is a matter of being tied to a theory which describes certain aspects of (physical) reality and may remain silent about others. From an essentialist perspective, the theory may thus not capture all physical aspects of the relevant system’s essence, but still *some* of them.

Picking up where the general discussion of Sect. 3.1 left off, this specificity of kinematical constraints could, however, turn out to conflict with our thesis in the following two kinds of cases: First, cases in which there are distinct (correct) physical theories which differ in whether at least one law expresses a kinematical constraint on the very same system. In cases of this kind, our thesis would seem to entail that this law both holds and fails to hold with metaphysical necessity, which is of course contradictory. Second, cases in which a theory is replaced by another one in which at least one law falls on the other side of the kinematical/dynamical distinction compared to the previous theory. In cases of this kind, our thesis would also seem to have the same problematic implication.

Again, the most straightforward and metaphysically conservative response to these two problem cases says that our thesis is only meant to apply to the laws of a unique, final physical theory (at least for a particular domain). Only those laws which belong to the kinematical structure of this final theory express metaphysical necessities. If this final theory exclusively covers its domain, neither of the two problem cases can arise and the tension between the fact that kinematical constraints are always kinematical constraints of a particular theory, and the metaphysical standard view that essence is absolute is resolved. The main drawback of the response is, again, that based on the current state of physics, this response may be seen as involving a (heavy) idealization about scientific progress. After all, while we can of course currently hope that we will at some point in time arrive at a unique, final physical theory, we neither have a guarantee that science will ever get there, nor can we make warranted assertions about the content of this theory. According to this response strategy, our appeal to particular examples of laws, including the recurring contrasting examples of the PEP and Coulomb’s law and all other examples given in this section, would have to be seen as merely tentative. Whether any of these really turn out to be metaphysically necessary laws of nature could, after all, only be settled based on whether they are laws of a final physical theory, a theory which we might not have or at least not know to have.

The second, metaphysically more revisionary response we mentioned in Sect. 3.1 has the advantage of allowing us to take current physical theories at their face-value: Its main idea is to match the theory-relativity of the kinematical/dynamical distinction on the side of essence: Entities have essences, but these essences are theory-relative. As a result, the claims we make about the metaphysical necessity of physical laws can be taken at their face value: We can hence assert, e.g. that the PEP expresses an essential truth about closed physical systems containing fermions (and about the ${\mathcal {N}}$-relation) *relative to certain quantum mechanical theories*.

The idea to relativize essence to a theory can be considered unorthodox, especially from the perspective of the dominant Neo-Aristotelian approach to essence made popular by Fine, Lowe, Correia and others,^53^ but it is not wholly without precedent. Lewis has famously suggested that what counts as essential to an entity is highly context-dependent (cf. Lewis (1986), p. 252) and Paul has developed a matching context-dependent theory of essence (cf. Paul 2004). On a different sort of relativist view, essence is *explanation*-relative, namely relative to an explanatory framework (see Sullivan 2017).

A metaphysically revisionary account along these lines could perhaps be adapted to deliver a theory-relative notion of essence. Another metaphysically revisionary strategy would consist in amending the Finean-approach to essence which we rely on in this paper to incorporate a relativized notion of essentiality; one would have to either adopt a matching relativized notion of metaphysical necessity in addition (cf. Hirèche 2020, 2021, Sect. 2.1), or develop a workable account of the connection between relativized essentiality and an absolute notion of metaphysical necessity. Spelling out the metaphysically revisionary accounts further is beyond the pale of this paper, so we provisionally adopt the first metaphysically *non-revisionary* reply as our official response here. In any case, we may conclude that, given the existence of workable ways to rule out problem cases arising from the theory-relativity of kinematical constraints, the ‘theory-relativity’ worry does not pose a serious threat to the essentialist DTA account.^54^

## Conclusion

Contemporary physics gives us reason to doubt the absolutist dogma propagated by the three dominant philosophical theories of laws of nature. Essentialist DTA gives us a non-absolutist theory of the laws—a theory which allows for both metaphysically contingent and necessary laws of nature—which is immune to the problems which plague two existing non-absolutist theories respectively proposed by Tahko, and by Hendry and Rowbottom. In particular, it meets the three crucial explanatory challenges for such theories which we have set out in this paper: First, it posits essence as the only source of necessity and explains the mere nomic necessity of the laws by defining this notion as a relativized notion of metaphysical necessity. Second, this also allows the theory to avoid the synchronization problem faced by a two-sources view, since it follows from the adopted definition of nomic necessity that metaphysically necessary laws are also nomically necessary. Third, the theory relies on Armstrong’s ${\mathcal {N}}$-relation as a distinct marker of laws of nature, allowing it to avoid the ‘disjunctification’ of the notion of a law which Bartels identified as a likely pitfall for non-absolutist views. We have furthermore argued that essentialist DTA can seamlessly be combined with a plausible criterion for distinguishing metaphysically necessary from metaphysically contingent laws of physics which draws on the distinction between kinematical and dynamical structure found in contemporary physical theories. Essentialist DTA thus gives us a theory of the laws of nature which, with respect to physics, meets naturalistic demands and at the same time improves on existing non-absolutist theories of the laws of nature.

## Data Availability

Not applicable

## Appendix: The omnipresence of the kinematical/dynamical distinction

In this appendix, we describe the presence of the kinematical/dynamical distinction across various spacetime theories, including our currently best spacetime theory (GR), and a candidate for our future best spacetime theory (canonical quantum gravity).

- **Newton-Cartan theory** In the Newton-Cartan framework (‘geometrised Newtonian gravity’)^55^, (A) a spacetime is characterised as the tuple $(M, t_{ab}, h^{ab}, \nabla _a)$ where
  - (i) *M* is a smooth, connected, four-dimensional differentiable manifold; (ii) $t_{ab}$ is a smooth, symmetric, covariant tensor field on *M* of signature (1, 0, 0, 0); (iii) $h^{ab}$ is a smooth, symmetric, contravariant tensor field on *M* of signature (0, 1, 1, 1); (iv) $\nabla _a$ is a smooth derivative operator on *M*; and (v) the following two conditions hold:
    $$\begin{aligned} \begin{aligned}&[...] h^{ab} t_{bc} = \mathbf{0 }\\&[...] \nabla _a t_{bc} = \mathbf{0 } \text { and } \nabla _a h^{bc} = \mathbf{0 } \end{aligned} \end{aligned}$$
    (Malament 2012, p. 249.)
  The conditions stated in the last two rows are called the “orthogonality” and “compatibility” condition, respectively. In other words, the kinematical set-up as a whole in terms of *h* and *t* fields is further restricted by the two kinematical constraints (v). Compare this to a narrower conception of classical spacetime according to which a (B) classical spacetime in addition has to be time-orientable, i.e., a classical spacetime is characterized as the tuple $(M, t_{ab}, h^{ab}, \nabla _a, \{\zeta ^a\})$ where $\{\zeta ^a\}$ denotes an equivalence class of time orientations. The signature of $t_{ab}$ implies that (vi) there is a covariant vector $t_a$ (unique up to sign) such that $t_{ab} = t_a t_b$. Here, (vi) is a derived kinematical constraint as it is uniquely (up to isomorphism) induced from the kinematical constraint that there is a class of time orientable vector fields. Prima facie, both (A) and (B) seem to be sensible conceptions of spacetime models. So why not think of the more restrictive (B) as the kinematical structure rather than just of the structure designated by (A) then? One argument to be made is that (A) might already be sufficiently restrictive for identifying spacetime structure given the background knowledge from general relativistic spacetime contexts that *global* time orientability is not at all necessary (as opposed to local time orientability) for a spacetime-object^56^—depending on whether or not we interpret the formalism on the basis of this background knowledge.^57^ This case then forcefully illustrates how the exact boundary between kinematical and dynamical structure depends on how we interpret the formalism behind a specific physical theory—in particular on the presupposed background knowledge through which the theory is interpreted—and thus underlines that the kinematical/dynamical distinction cannot be read out purely formally.
- **General relativity** In general relativity (GR), a spacetime is characterised by the tuple $(M, g_{ab})$ where *M* is a four-dimensional differentiable manifold, and *g* a Lorentzian metric. Prior to the actual dynamical equations of motion for the metric field—the Einstein field equations—various sorts of constraints can be imposed on the set of allowed spacetimes. Note that, for instance, a similar ambiguity as to whether or not a spacetime has to be time-orientable arises as in Newton-Cartan theory above. Independently of such quarrels, an example of a *derivative* kinematical constraint straightforwardly arises from the mere choice of the metric field *g* as a configurative variable: “every neighbourhood in every model spacetime admits coordinates (at least locally) in which the component of the scalar density [namely, $[\sqrt{-g}]$] has a value of $-1$” (Pitts 2006, p. 95).^58^
- **Canonical quantum gravity** Non-perturbative canonical quantisation of gravity takes in a Hamiltonian description of GR; this means that GR, first of all, needs to be constrained to its 3+1/ADM/formulation, that is formulated as a dynamical evolution of the metric constrained to a space-like surface $$\varSigma $$.^59^$$^{\text {,}}$$^60^ The presentation of canonical quantisation of GR in ADM formalism and Ashtekar-Barbero variables—quantising this choice of variables is sometimes called quantum geometry—closely follows Wüthrich (2006). The Hamiltonian in the ADM-formalism is given as $$H = \int _{\varSigma } {\mathcal {H}} d^3 x$$ with Hamiltonian density $${\mathcal {H}} = \sqrt{q} (N C + N^a V_a)$$ where $${C := -^{(3)} R + q^{-1} \pi ^{ab} \pi _{ab} - \frac{1}{2} q^{-1} \pi ^2}$$, and $$V_a := - 2 D^b (q^{-1/2} \pi _{ab} )$$. Here, $$q_{ab}$$ is the three-metric, *q* the determinant of $$q_{ab}$$, $${{{}^{(3)}} R}$$ is the 3-dimensional scalar curvature, $$\pi _{ab}$$ the canonical momentum, $$\pi = \pi ^a_a := \pi ^{ab} q_{ab}$$, $$D^a$$ is the unique torsion-free covariant derivative operator on $$\varSigma $$ associated with the three metric $$q_{ab}$$, and *N* and $$N^a$$ are respectively the lapse function and the shift vector central to the ADM formulation. The Hamiltonian *H* is decomposes into an orthogonal component $$H_\text {orth.} := {\mathcal {C}}(N) := \int d^3 x \sqrt{q} N C \approx 0$$, which vanishes if the equations of motion for *N* are imposed ($${\mathcal {C}}$$ is called the ‘Hamiltonian constraint’), and a parallel component $$H_\text {parallel} := {\mathcal {V}}(N^a) := \int _{\varSigma } d^3 x \sqrt{q} N^a V_a$$ which vanishes when the (formally dubbed) ‘equations of motion’ for $$N^a$$ are imposed ($$V^a$$ are called the ‘spatial diffeomorphism constraints’). *N* and $$N^a$$ can respectively be seen to act as Lagrange multipliers.^61^$${\mathcal {C}}(N)$$ and $${\mathcal {V}}(N^a)$$ together form an algebra named Dirac algebra, which is schematically of the following form:
  $$\begin{aligned}&\{{\mathcal {V}}, {\mathcal {V}}\} \propto {\mathcal {V}},\\&\{{\mathcal {V}}, {\mathcal {C}}\} \propto {\mathcal {C}},\\&\{{\mathcal {C}}, {\mathcal {C}}\} \propto {\mathcal {V}}. \end{aligned}$$
  The constraints are so-called first-class constraints, that is, their mutual Poisson brackets vanish when the constraints are imposed. The canonical commutation relations are ${\{ q_{ab} (x), \pi ^{cd} (y) \} \propto \delta {{}^{3c}_{(a} } \delta {{}^{3d}_{b)} } (x, y)}$, and $\{q_{ab} (x), q_{cd} (y)\} = \{\pi _{ab} (x), q_{cd} (y)\} = 0$. Standard canonical quantum gravity straightforwardly quantises this Dirac algebra using Dirac’s method of constrained quantisation; canonical loop quantum gravity analogously quantises the extended Dirac algebra obtained after a change to Ashtekar variables through the same method.^62^ The general procedure runs as follows:^63^
  - Find a space of functionals ${\mathcal {K}}$ to represent the operators corresponding to the canonical variables.
  - Impose all constraint equations apart from the Hamiltonian constraint onto ${\mathcal {K}}$, that is, the constraints related to the parallel component of the Hamiltonian. The space of solutions provides a subspace ${\mathcal {M}} \subset {\mathcal {K}}$. Find an inner product for turning ${\mathcal {M}}$ into a Hilbert space. (Alternatively, find a suitable inner product for ${\mathcal {K}}$ which can then be inherited by ${\mathcal {M}}$.) The resulting Hilbert space is called the kinematical Hilbert space ${\mathcal {H}}_{kin}$. It is the space obtained through realising all kinematical constraint equations. This step thus amounts to imposing kinematical constraints.
  - Impose the Hamiltonian constraint. The resulting Hilbert space is called the ‘physical Hilbert space ${\mathcal {H}}_{phy}$’.
  All expectation values for evolution between two ‘slices’ of spacetime can now be calculated through forming the inner product of states in ${\mathcal {H}}_{phy}$. It is worth noting that, *after* quantisation, states in the kinematical Hilbert space are usually re-expressed in the so-called spin network representation. We can make the following observations on kinematical constraints: (1) The conditions literally dubbed ‘kinematical constraints’ here (there are others prior to them, such as resulting from choosing a $3+1$ split in the first place) are prior to the dynamical constraint impositions. (2) However, it is not unthinkable to solve first the dynamical constraints and then the so-called kinematical constraints. (Generally speaking, ‘fixing gauge’, and ‘imposing dynamics’ commute.) (3) It is because one wants to know the kinematical states (and also because one can practically achieve this) that one is already interested in the kinematical Hilbert space. (4) If one had solved the dynamical constraints first, it would be the dynamics of a much more general theory with much more general states whose meaning we would presumably not understand (what systems would they refer to?) than that of the kinematical Hilbert space for which we have solved the kinematical albeit not the dynamical constraints. Crudely speaking, kinematics is essential for knowing *what we are talking about*, dynamics for knowing how *what we are talking about* evolves.

## Footnotes

1 Though arguably the most popular options, those three approaches are not the only ones available. In particular, while representing very different overall accounts of laws (e.g. as regards modal status), all three are *reductionist* accounts; yet, there are also primitivist views (e.g. Carroll 1994; Maudlin 2007), and eliminativist views (for two eliminativist views based on radically different motivations, see van Fraassen 1989b, and Mumford 2005).

2 Although primitivism about laws does not seem to imply any answer to the modal question—in principle, a primitivist could be a necessitarian or a contingentist, or even a non-absolutist—, the main existing primitivist views are also absolutist, taking all laws to be *contingently* existing/obtaining entities/facts that cannot be further metaphysically accounted for in terms of something else (Carroll 1994; Maudlin 2007).

3 This formulation—and similar formulations to follow—is to be understood in the sense of “in virtue of the nature of the *natural kind* fermions”, not as ‘in virtue of the nature of individual fermions”.

4 This point about the PEP may appear to be at odds with the previous claim that Coulomb’s law is metaphysically contingent: Why should Coulomb’s law not be taken to hold just in virtue of the nature of Footnote 4 continued electrons and other charged particles? Whereas Tahko (2015) motivates the claimed difference in modal status merely intuitively (based on whether a law features a fundamental natural kind), we will show in Sect. 3 that it can in fact be accounted for based on the different statuses that those two laws have within physical theorising itself.

5 This would make, using Hale’s terminology, metaphysical necessity at least as strong as, and indeed stronger than, nomic necessity. According to Hale (1996), p. 94, a kind of necessity $\Box _1$ is stronger than a kind of necessity $\Box _2$ if, and only if, for all *p*, $\Box _1 p \rightarrow \Box _2 p$, but not for all *p*, $\Box _2 p \rightarrow \Box _1 p$. Dispositional essentialists, in particular, reject the idea that metaphysical necessity is stronger in this sense than nomic necessity, since they identify the two kinds of necessity.

6 See Tahko (2015), pp. 516f, 524f.

7 One can arguably read Tahko’s view in a more epistemic fashion: the best-system view, unlike an essentialist view, of the laws is then not seen as accounting for the ‘objective’ laws of nature, but rather providing Footnote 7 continued (epistemic, practical) criteria for us to best systematize the regularities in the world. This would mean that Tahko’s view does not immediately face the synchronization problem, which is a purely metaphysical worry (likewise for the disjunctiveness concern mentioned below). Note that this reading of the best systems account (*BSA* for short) as epistemic in nature is non-standard, since it does not give us the deflationary *ontic* account of laws delivered by the standard version of the BSA, and that we take Tahko indeed to be interested in an ontic hybrid law account. Note also that this epistemic take on the BSA would leave the metaphysical question about the ontic status of the laws unanswered. Thanks to Valentina Luporini for pointing this out to us.

8 Slightly more precisely: that a pair of a hydrogen atom which is covalently bonded to an electronegative atom in a molecule and another electronegative atom in the same or another molecule may enter into electrostatic dipole-dipole interaction.

9 See (Lewis 1968, 1986).

10 Note that, as Hendry and Rowbottom (2009), pp. 674-5 point out, this does not mean that dispositional contextualists are forced to follow along a sorites series of ever so slightly different dispositional profiles which leads them into paradox (cf. Hyde and Raffman 2018). A sorites series of this kind could, for example, start with the claim that water has its actual dispositional profile with respect to the actual world and proceed, step-by-step, to the claim that relative to some other possible worlds, it has the dispositional profile of a completely different substance, e.g. that of sodium chloride. The intransitivity of the counterpart relation guards their theory against this rather disastrous outcome.

11 Since Hendry and Rowbottom explicitly take dispositional essentialism as their starting point, we assume that their theory inherits the dispositional essentialists’ assumption that there is at least one property which supports the truth of a law with respect to all possible worlds in which it holds. In theory, they could also allow laws which are supported by some property in some worlds and by others in other worlds.

12 To be more precise, the profile is constant with respect to those of its dispositional features connected to the law. If there are any, other dispositional features of the property which are unconnected to the law may of course still vary. The same holds for metaphysically contingent laws with respect to the set of the nomically possible worlds. We will leave these specifications implicit in the following when mentioning constant dispositional profiles.

13 Cf. the discussion of Chisholm’s paradox and the essentiality of origin in Lewis (1986), pp. 243ff. See also Paul (2004) for discussion.

14 There is of course more to be said about this sort of criterion. For example, one important question about it is which sorts of dispositional properties support laws of nature in the suggested way. A natural idea would be to assume that only perfectly natural properties play this role.

15 But note that there is a recently growing discussion of metaphysical indeterminacy which questions the orthodoxy. See e.g. Barnes (2010), Calosi and Mariani (forthcoming), Smith and Rosen (2004), and Wilson (2013).

16 We follow Tooley in taking the laws of nature to be universal generalizations which are due to the *N*-relation, rather than the states of affairs of the *N*-relation holding between universals themselves. Armstrong and Dretske adopt the latter view, by contrast. Universals still have an important role to play in our theory, however (see Sect. 2.2).

17 In what follows, we use letters such as ‘${\mathcal {N}}$’, ‘${\mathcal {F}}$’ and ‘${\mathcal {G}}$’—as opposed to the predicate-letters ‘${\mathcal {N}}$’, ‘*F*’ and ‘*G*’—as singular terms for properties.

18 In our discussion, we focus on deterministic laws for the sake of simplicity. The account could be generalized to also capture probabilistic laws, however. In the case of a probabilistic law, we have various possible outcomes $G_{1}x, G_{2}x$ etc. that follow from *Fx* with certain conditional probabilities $p_{1}, p_{2},..$. Footnote 18 continued To account for such laws, one might, for instance, postulate a multiplicity of *N*-relations that each has an associated conditional probability—which, in the limit case of a deterministic law equals 1. Then, we could model the case of a probabilistic law as one in which ${\mathcal {F}}$ stands in ${\mathcal {N}}_{1}$ to ${\mathcal {G}}_{1}$, in ${\mathcal {N}}_{2}$ to ${\mathcal {G}}_{2}$ etc.

19 Another important problem is the *identification problem* (see van Fraassen 1989a, p. 97): What does the ${\mathcal {N}}$-relation consist in? In general, the question of what sort of relation ${\mathcal {N}}$ is supposed to be has so far received no precise and convincing answer. We will provide one through our essentialist DTA-account below though.

20 See, for instance Armstrong (1983), Tooley (1987), Forge (1986) and Maudlin (2007). Tooley in particular provides a solution to the form of the law problem which could serve as an alternative to the solution which we will now propose.

21 One might worry that this account has it that quite complex—and thus presumably non-natural/non-fundamental—properties are taken to be ${\mathcal {N}}$-related and to thus figure in the (sources of) the physical laws. However, we can think of no way of rendering this objection more precise that strikes us as compelling. First, pace the assumption that such complex properties do not even exist or are no ‘genuine’ properties, see e.g. Bealer (1982) and Bealer and Mönnich (1989). Second, we take Hicks and Schaffer (2015) to have ruled out the idea that only fundamental properties can feature in the laws of physics. And third, in response to the worry that some N-facts are plausibly fundamental and that our proposal thus conflicts with Sider’s purity principle—according to which only fundamental entities can figure in fundamental facts—we would like to point out that the purity principle is itself contentious and faces serious difficulties (see e.g. Merricks (2013)). While a more thoroughgoing defense of the proposed move would be certainly preferable, we have to leave this to another occasion. The reader who is not convinced by the considerations in this footnote, however, should feel free to adopt one of the other alternatives regarding the form of the laws challenge, such as those proposed by Armstrong (1983) or Tooley (1977).

22 In direct analogy to Friend (2016), p. 134: “Newton’s law of gravitation is a conditional which states that all two-mass systems will experience gravitational components of force proportional to the product of each other’s mass according to the equation $F_M= F_m = G \frac{M m}{r^2}$.”

23 In particular, Armstrong (1983)’s solution, according to which the ${\mathcal {N}}$-states of affairs are at the same time universals, has the disadvantage that, as Armstrong puts it himself, “Logicians are inclined to protest on this point.” (p. 90). For objections to Tooley’s speculative solution, see Sider (1992).

24 One may distinguish two senses in which (Necessary Implication) might be regarded as a ‘fair stopping point’: The first is that of a *metaphysical* stopping point, i.e., a brute truth that simply lacks any deeper metaphysical explanation. If (Necessary Implication) was such a legitimate stopping point on this understanding, no further metaphysical account of why it holds *could* be given even in principle—there would simply be nothing more which could be said. The second kind of a legitimate stopping point, by contrast, would be a *methodological* one, i.e., a stopping point just because one has to stop somewhere (the foundationalist’s dilemma) and which is, nevertheless, acceptable in terms of philosophical *theory building*. If (Necessary Implication) were a legitimate stopping point thus understood, its proponents would not need to offer a further explanation of why it holds, even though such an explanation *could* potentially be given; they would simply be free to refrain from saying more.

25 Which means that our account differs from the absolutist account presented in Swoyer (1982) which relies on the essences of properties, not the essence of the ${\mathcal {N}}$-relation.

26 See Fine (1994) for the idea that modality is analysable in terms of essence rather than the other way around, and Fine (1995), Correia (2012) and Michels (2019) for discussions of the reduction of metaphysical modality in terms of essence.

27 An essentialist solution along these lines is already hinted at in Barker and Smart (2012). They reject it, however, apparently based on the idea that, (a), the essentialist claim would have it that the two facts [$N(\mathcal {F,G})$] and [$\Box \forall x (Fx \rightarrow Gx)$] are identical, and (b), the proponent of the DTA account should not take these facts to be identical (pp. 720f.). But while we are happy to grant (b), we can see no good reason to endorse (a).

28 Furthermore, (Essence of N) also provides an answer to the related identification problem of the DTA account. (Cf. again van Fraassen 1989a, p. 97.) In fact, it is unsurprising that our proposed view provides an answer to both problems at the same time. For on this view, what the ${\mathcal {N}}$-relation is amounts to what it does: the ${\mathcal {N}}$-relation just is the relation that makes the instances of its first relatum always come with instances of its second relatum. Note, in particular, that this answer to the identification problem avoids the difficulties faced by Armstrong’s suggestion that N may be identified with ‘the causal relation’ (see Armstrong 1993, p. 422.): on our view, causation can perfectly well be understood as a relation between concrete particulars—rather than between (both concrete particulars and) universals –, and the resulting account of the laws is perfectly compatible with there being causal as well as non-causal laws.

29 Note that incidentally, Armstrong himself uses the essentialist form of words ‘what it is to be a...’, which led Aristotle’s Roman translators to coin the term ‘essence’ in the first place, to explain what the source of the necessity of the laws is: ‘We are now saying that, for it to be a law that an *F* is a *G*, it must be necessary that an *F* is a *G*, in some sense of ‘necessary’. But what is the basis in reality, the truth-maker, the ontological ground, of such necessity? I suggest that it can only be found in what it is to be an *F* and what it is to be a *G*.’ (Armstrong 1983, p. 71.)

30 We are assuming the rather common view that essence is monotonic: if a proposition $\varPhi$ is true in virtue of the essence of some plurality of entities *xx*, and *yy* is a plurality that contains *xx*, then $\varPhi$ is also true in virtue of the essence of *yy* (see Fine 1995; Zylstra 2019). However, one may have independent reasons to think that essence is non-monotonic (see Michels 2018). And if such a view were assumed, (Mixed Essential Holding), in its current form, may be problematic: In particular, we should not exclude the possibility that, for some or indeed all metaphysically necessary laws, the ${\mathcal {N}}$-relation holds in virtue of the essence of only *one* of the universals involved. For instance, it is arguably a metaphysically necessary law that fermions cannot share the same quantum numbers (PEP), but it is plausible that the ${\mathcal {N}}$-relation between the property of being a two-particle fermion system and the property of the system that the constitutive fermions display different quantum numbers holds in virtue of the essence of the *former* property alone. Thus, if we wanted to make our account compatible with a non-monotonic understanding of essence, we would have to modify (Mixed Essential Holding). Yet, the relevant modification would be minor, leaving the core ideas behind our account untouched. We would just have to replace (Mixed Essential Holding) with the slightly more complex (Mixed Essential Holding*): $\exists {\mathcal {X}}_{1}, {\mathcal {Y}}_{1} (N({\mathcal {X}}_{1},{\mathcal {Y}}_{1}) \; \& \; \lnot \exists xx \Box _{xx} N({\mathcal {X}}_{1},{\mathcal {Y}}_{1})) \; \& \; \exists {\mathcal {X}}_{2}, {\mathcal {Y}}_{2} \; ( \Box _{{\mathcal {X}}_{2}, {\mathcal {Y}}_{2}} N({\mathcal {X}}_{2},{\mathcal {Y}}_{2}) \; \vee \; \Box _{{\mathcal {X}}_{2}} N({\mathcal {X}}_{2},{\mathcal {Y}}_{2}) \; \vee \; \Box _{ {\mathcal {Y}}_{2}} N({\mathcal {X}}_{2},{\mathcal {Y}}_{2}))$.

31 Bird (2005) claims that the conflict with quidditism already arises as soon as (Necessary Implication) is endorsed. But as Coates (2019) points out, the conflict only arises if the essentialist claim is endorsed.

32 This simple account (see Hale 1996 for the standard version according to which nomic necessity is a relativized form of *logical* necessity) has faced objections. Two of them are due to Fine (2002) (see Leech 2016 for responses). First, defining necessities by relativization, one may end up with ‘cheap’ kinds of necessities—e.g. the necessities about your desk, defined as the propositions metaphysically/logically entailed by the basic truths about your desk. As Leech (2016) (Sect. 2.2) notes in response, however, we can reasonably distinguish between necessities that are indeed “cheap” in this sense and necessities that are *not*, because the corresponding relativization basis is not purely arbitrary but clearly significant. On her view, such a significant relativization basis for nomic necessity is given by the laws of nature themselves; on our view, it is given by proposition $\varPhi$, the conjunction of all ${\mathcal {N}}$-*propositions* (i.e. propositions of the form $<{\mathcal {N}}$ holds between ${\mathcal {F}}$ and ${\mathcal {G}}>$5). A second worry is that, on the standard relativization account, the laws of nature come out as nomically necessary *trivially*, by mere self-entailment: For every conjunct $\lambda \in \varPhi$, we have $\boxminus \lambda$, by the logical form of the definition. Yet, the objection goes on, one would expect the reason for a law’s being nomically necessary to amount to something more substantial and not just to a trivial logical implication of the definition of nomic necessity. It is worth noting that our proposed account does not face this worry (in the same way): on our view, laws themselves are not nomically necessary by mere self-entailment, but because they are entailed by the ${\mathcal {N}}$-*propositions*, and this entailment has a metaphysically more substantial explanation based on essence which is captured by our principle Essence of ${\mathcal {N}}$. More recently, other objections have been addressed by Bob Hale and Jessica Leech (Hale and Leech 2017). We will not discuss them here; suffice it to say that Hale and Leech propose a refined version of their definition of nomic necessity which avoids all those worries: $\boxminus p := \exists q_1, \ldots , q_n (\varPi (q_1), \ldots ,\varPi (q_n)) \; \& \; \Box ((q_1 \& \ldots \& q_n) \rightarrow p)$, where $\Box$ is the logical necessity operator, and “$\varPi (q)$” may be read as ‘It is a law of physics that *q*’. To avoid those same problems, we could endorse the same refined definition, except that $\Box$ would be the *metaphysical* necessity operator, and “$\varPi (q)$” would stand for ‘q is an ${\mathcal {N}}$-*proposition*’. On such an account, it may no longer be the case that all metaphysical necessities are automatically nomic necessities (see Hale and Leech 2017, Sect. 7). It will be the case if, and only if, there is at least one ${\mathcal {N}}$-proposition. Yet, this poses no problem for our account. First, what really matters, for our account to avoid the synchronization problem, is that all metaphysically necessary *laws* are nomically necessary. And this cannot fail to be the case on our view: if there is any metaphysically necessary law, then there will be a corresponding ${\mathcal {N}}$-proposition. Second, in the context of our discussion, it is anyway tacitly assumed that there *are* at least some laws (given non-absolutism, at least two), and thereby at least some ${\mathcal {N}}$-propositions.

33 See Wolff (2013) and Linnemann (2020) for discussion.

34 Note that this gives us a response to the problem for non-absolutist accounts raised in Shoemaker (1980), section IX, that of them not being able to provide a principled way to distinguish between nomically and metaphysically (‘logically’ in Shoemaker’s terminology) necessary laws. Our response is based on the idea that the criterion Shoemaker asks for can only be provided by reasoning informed by the structure of physical theories.

35 We take it to be uncontroversial that all physical laws are at least nomic necessities.

36 Arguments against the view have for example been given in MacBride (1999), Fritz (2017), and Wang (2016).

37 See e.g. the discussion of light and heat in Kripke (1980), p. 128.

38 See e.g. Fine (1994), Lowe (2008), Correia (2006).

39 We take the omnipresence of the kinematical/dynamical distinction in physical theories—which is argued for explicitly in a subsequent section and the appendix—to strongly suggest that a putative final theory will showcase such a distinction just as well. Needless to say, we will not be able to avoid the pessimistic charge by McKenzie (2019) that the extrapolation might fail—but no naturalised metaphysics project interested in fundamental ontology can.

40 There might be an alternative which strikes a compromise between the two mentioned positions. Bartels proposes to relativize essences to explanatory goals instead of theories, where an explanatory goal is pursued by scientists in a cumulative succession of increasingly precise theories, each providing us with a more precise picture of what the essences of entities in our world are. According to this view, essence, and thereby the notion of metaphysical necessity, is relativized to a larger explanatory project, but not to a specific theory within that project. See Bartels (1996), Sect. 2. We are grateful to an anonymous reviewer for drawing our attention to this view.

41 The distinction arguably resembles Earman’s ‘constraint vs. dynamical equation’ distinction (see Earman 1995). Thanks to an anonymous reviewer for pointing this out to us. Although we follow Curiel here (see also next section), at the end of the day our metaphysical proposal is independent of whether the distinction from physics in question is called the ‘kinematical/dynamical’ or the ‘constraint/dynamical’ distinction.

42 Cf. Curiel (2016).

43 Needless to say, these two notions of kinematics are not unrelated: everything one does in $\hbox {kinematics}_{\text {discipline}}$, that is, analyse *what* describes motions and their *constraints* is concerned with the $\hbox {kinematics}_\text {kinematical structure}$ of a physical theory.

44 More precisely, we need to be clear about our best physical theories qua *interpreted formalisms*. This being said, the formalism of a physical theory should be taken seriously as a guide towards an interpretation in the first place. Otherwise, we are always at danger of baking strong metaphysical presuppositions into the theory to begin with—thereby making the project of naturalised metaphysics overly circular.

45 See Linnemann and Martens (2021) for an extended argument to this effect.

46 Note that an epistemic (or, more specifically, transcendentalist) interpretation of the kinematical-dynamical distinction is possible as well: Curiel himself, for instance, makes the allusion to kinematic constraints as a relativised a priori in the spirit of Reichenbach (1965), or Friedman (2001). Just as before where we dismissed a merely epistemic reading of Tahko’s law account (see 7), we, however, only take interest in the more straightforward ontic reading (in other words, we are interested in a project of naturalised, realist ontology).

47 An insightful heuristic treatment of such proofs is provided by Baez (2012); an in-depth discussion by Bain (2016).

48 Bain (Unknown) for instance arrives at the similar conclusion that the spin-statistics theorem is kinematical in nature.

49 See Lange (2009) for a view on which there are various grades of nomic necessity corresponding to degrees of stability under counterfactual variation.

50 It is important to emphasise that Curiel takes all of this to eventually speak in favour of a transcendentalist reading of kinematical constraints: Kinematical constraints are then constitutive of physical theories in the narrower sense that they express the minimal preconditions relative to which specific physical content can be meaningfully expressed and interpreted. As kinematical constraints differ from theory to theory, this transcendentalist take on kinematical structure is naturally related to the relativised a priori of Reichenbach (1965) and Friedman (2001) (as Curiel stresses himself). Note that none of this here is a problem for our view as we are simply working under the different proviso that current non-transcendentalist analytic metaphysics—naturalised or not—is the right way to go; and as clearly the kinematical/distinction cannot settle the debate on whether one can sensibly or not speak of the things in themselves, it is simply subject to our general metaphysical leanings whether the kinematical/dynamical distinction supports a non-absolutist law account or a transcendentalist reading. This being said, *both* options are of course prima facie on the table; this paper only explores one out of the two. A detailed exploration of the kinematical/dynamical distinction and of theory interpretation in physics from a transcendentalist position more generally is arguably Curiel (2020).

51 This characterisation goes back to Aristotle, see his *Metaphysics Z.4* for the ‘what it is to be’-formulation and *Topics 102a3* for the link to definition. The latter aspect in particular has been stressed in the context of contemporary essentialism by Fine (1994).

52 We thank an anonymous referee for pushing us to clarify this aspect of our argument.

53 See e.g. Correia (2012), Fine (1994), Lowe (2018).

54 The alternative approach advocated in Bartels (1996), Sect. 2 mentioned in footnote 40) again promises to offer a middle way: If essence is relativized to an explanatory project instead of to a specific theory and if such explanatory projects comprise a succession of ever more precise theories with the same explanatory goals, the kinematical constraints of a theory, and with them the essential truths they express about physical systems, are refined, not exchanged as we progress through this succession. Essence and metaphysical necessity are hence still relativized, but in a less pervasive manner, given that a large number of physical theories may contribute to the same large-scale explanatory project.
